# Turning a Negative into a Positive: Ascending GABAergic Control of Cortical Activation and Arousal

**DOI:** 10.3389/fneur.2015.00135

**Published:** 2015-06-11

**Authors:** Ritchie E. Brown, James T. McKenna

**Affiliations:** ^1^Laboratory of Neuroscience, Department of Psychiatry, VA Boston Healthcare System, Harvard Medical School, Brockton, MA, USA

**Keywords:** wakefulness, gamma rhythm, theta rhythm, EEG, arousal, hypnotics

## Abstract

Gamma-aminobutyric acid (GABA) is the main inhibitory neurotransmitter in the brain. Recent technological advances have illuminated the role of GABAergic neurons in control of cortical arousal and sleep. Sleep-promoting GABAergic neurons in the preoptic hypothalamus are well-known. Less well-appreciated are GABAergic projection neurons in the brainstem, midbrain, hypothalamus, and basal forebrain, which paradoxically promote arousal and fast electroencephalographic (EEG) rhythms. Thus, GABA is not purely a sleep-promoting neurotransmitter. GABAergic projection neurons in the brainstem *nucleus incertus* and *ventral tegmental nucleus of Gudden* promote theta (4–8 Hz) rhythms. *Ventral tegmental area* GABAergic neurons, neighboring midbrain dopamine neurons, project to the frontal cortex and nucleus accumbens. They discharge faster during cortical arousal and regulate reward. *Thalamic reticular nucleus* GABAergic neurons initiate sleep spindles in non-REM sleep. In addition, however, during wakefulness, they tonically regulate the activity of thalamocortical neurons. Other GABAergic inputs to the thalamus arising in the *globus pallidus pars interna, substantia nigra pars reticulata*, *zona incerta, and basal forebrain* regulate motor activity, arousal, attention, and sensory transmission. Several subpopulations of cortically projecting GABAergic neurons in the *basal forebrain* project to the thalamus and neocortex and preferentially promote cortical gamma-band (30–80 Hz) activity and wakefulness. Unlike sleep-active GABAergic neurons, these ascending GABAergic neurons are fast-firing neurons which disinhibit and synchronize the activity of their forebrain targets, promoting the fast EEG rhythms typical of conscious states. They are prominent targets of GABAergic hypnotic agents. Understanding the properties of ascending GABAergic neurons may lead to novel treatments for diseases involving disorders of cortical activation and wakefulness.

## Introduction

Our current understanding of the brain and the mechanisms involved in switching between different behavioral states is based on the investigational tools available to researchers. Thus, the development of histochemical and immunohistochemical methods to identify monoaminergic and cholinergic neurons, together with biochemical methods to study their metabolism, led to the concentration of neuropsychiatric research on these neurotransmitter systems in the latter part of the 20th century (1). Similarly, in the sleep-wake field, much research focused on the role of the monoaminergic and cholinergic neurotransmitter systems (2), leading to an influential theory on the mechanisms which control switching between non-REM and REM sleep (3, 4). By contrast, much less attention was paid to the control of the sleep-wake cycle by the more prevalent gamma-aminobutyric acid (GABA)ergic and glutamatergic systems due to their later discovery and the lack of tools to manipulate and record their activity.

The sleep-promoting effects of allosteric agonists of the GABA_A_ receptor are well-established (5). However, we have only recently discovered the location and properties of GABAergic neurons controlling the sleep-wake cycle. The sleep-promoting action of GABAergic neurons located in the preoptic hypothalamus (6–8) is now well-known and accepted (9). More recently, other groups of sleep-promoting GABAergic neurons in the lateral hypothalamus (melanin-concentrating hormone neurons) and brainstem [parafacial zone; (10)] have been identified. It is less well-appreciated that there are several groups of fast-firing, subcortical GABAergic neurons with ascending projections, which promote wakefulness and cortical activation. Given that GABA is normally an inhibitory neurotransmitter in the adult brain, this promotion of cortical activation may seem paradoxical. However, the important concept to be grasped is that these ascending GABAergic neurons do not exert a tonic inhibition of their cortical and thalamic targets, but rather, they sculpt the pattern of activity of their targets in favor of the fast oscillations of firing typical of brain-activated states. Here, we summarize our current knowledge of these neurons, which recent evidence suggests may be critical in promoting cortical activation during wakefulness and REM sleep.

We first describe the technological advances that have allowed investigation of these GABAergic cell populations, followed by a review of the properties and likely functions of each group of GABAergic neurons.

## Novel Technological Tools have Allowed Us to Identify, Record from, and Selectively Manipulate the Activity of GABAergic Neurons Controlling the Sleep-Wake Cycle

In the last two decades, rapid advances in technology have allowed us for the first time to selectively identify and manipulate the activity of GABAergic neurons involved in control of the sleep-wake cycle. These techniques are essential since the GABAergic neurons involved in these processes are often interspersed among other neurons utilizing different neurotransmitters. *Immunohistochemical staining for the enzyme which synthesizes GABA, glutamic acid decarboxylase (GAD)*, or staining for GABA itself, allowed the precise study of the distribution of GABAergic neurons in sleep-wake controlling regions of the brainstem, hypothalamus, and basal forebrain (BF) (6, 11–16). When combined with *Fos immunohistochemistry* to stain the nuclei of neurons which were recently active (i.e., expressed this immediate-early gene product), these immunohistochemical techniques proved invaluable in revealing the location of sleep- or wake-active GABAergic neurons (13–15).

While useful in determining the location of wake-active GABAergic neurons, Fos immunohistochemistry cannot reveal their firing rate or pattern, due to its limited temporal resolution. For this, electrical recordings in intact animals are essential. Heroic *juxtacellular unit recordings in anesthetized and awake rats* allowed characterization of the discharge of identified GABAergic neurons across the sleep-wake cycle for the first time (17–20). In this technique, recordings of extracellular action potentials from individual neurons are performed with glass micropipettes containing neurobiotin, which enters the recorded neuron, allowing subsequent immunohistochemical identification using co-staining for GAD or other markers such as parvalbumin (PV), a calcium binding protein expressed in subsets of fast-firing GABAergic neurons in many brain areas (21–24).

**Figure 1:**
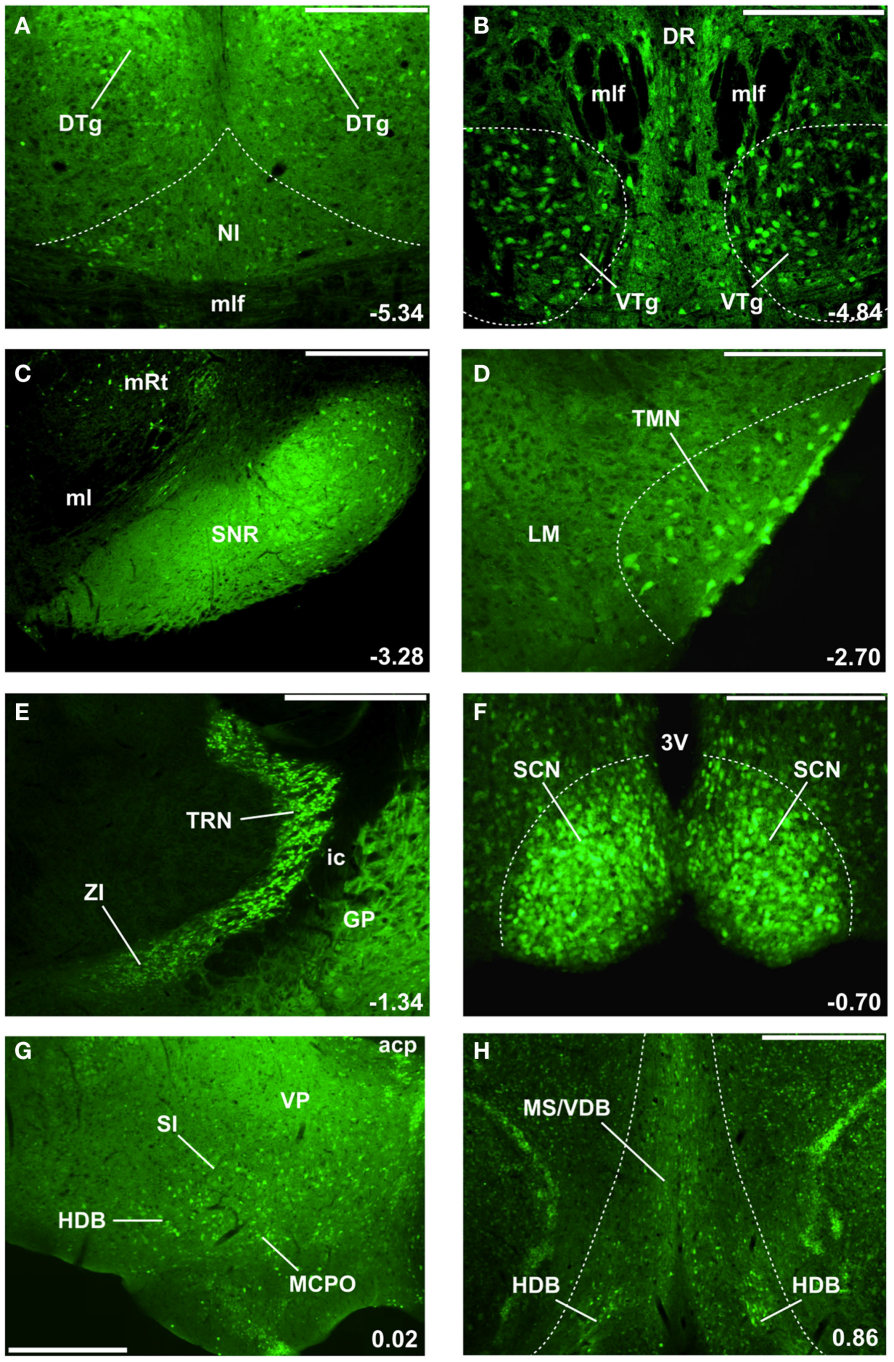
**GAD67-GFP knock-in mice delineate the location of GABAergic neurons involved in cortical arousal and sleep-wake control** (24, 25, 28, 34). The location of select nuclei described in this review is illustrated, moving in a caudal to rostral direction through the mouse brain **(A–H)**. Numbers in the bottom right corner indicate the location of these coronal sections with respect to Bregma (in mm). Green (GFP) fluorescence is present in cell bodies and fibers of GABAergic neurons. **(A)** Nucleus incertus (NI) GABAergic neurons involved in theta-rhythm generation are located near the midline of the central gray, above the medial longitudinal fasciculus (mlf), and ventromedial to the densely packed GABAergic neurons in the dorsal tegmental nucleus of Gudden (DTg). DTg neurons are involved in signaling head direction and project to the lateral mammillary body. **(B)** Ventral tegmental nucleus of Gudden (VTg) GABAergic neurons are clustered ventral to the mlf and the dorsal raphe (DR) nucleus. They innervate glutamatergic neurons in the medial mammillary body (see Figure 2). **(C)** GABAergic cell bodies and fibers delineate the substantia nigra pars reticulata (SNr), located lateral to the medial lemniscus (ml) and medial reticular nucleus (mRt). SNr GABAergic neurons represent the main output of the basal ganglia in rodents and tonically inhibit the motor thalamus and the centromedian-parafascicular nucleus (CM-Pf). **(D)** Tuberomammillary (TMN) histamine neurons located lateral to the lateral mammillary nucleus (LM) also express GABAergic markers and may release GABA. **(E)** Thalamic reticular nucleus (TRN) GABAergic neurons surround and inhibit almost all thalamic relay nuclei. More ventrally and medially are the zona incerta (ZI) GABAergic neurons, which project to higher-order thalamic nuclei and the neocortex. The internal capsule (ic) separates these nuclei from GABAergic neurons in the globus pallidus (GP). **(F)** The master circadian pacemakers in the suprachiasmatic nucleus (SCN) of the hypothalamus are GABAergic. 3V = 3rd ventricle. **(G)** Caudal/intermediate nuclei of the basal forebrain contain many large-sized GABAergic neurons which project to the neocortex and regulate gamma oscillations and wakefulness. acp, anterior commissure, posterior part; HDB, horizontal limb of the diagonal band; MCPO, magnocellular preoptic nucleus; SI, substantia innominata; VP, ventral pallidum. **(H)** Rostral nuclei of the basal forebrain, the medial septum (MS), and vertical limb of the diagonal band (VDB) contain GABAergic septohippocampal neurons regulating hippocampal theta and gamma rhythms. Scale bars: **(A,B,D,F)** 0.25 mm; **(C,G,H)** 0.5 mm; **(E)** 0.75 mm.

Extracellular unit recordings *in vivo* are essential for establishing the normal firing patterns of GABAergic neurons and correlating them with the electroencephalographic (EEG) activity and behavior. However, they have a limited ability to reveal the underlying cellular mechanisms, i.e., the ion channels and neurotransmitter receptors which are the targets of most pharmacological agents used clinically to modulate brain activity. To determine these cellular mechanisms, *intracellular recordings of identified GABAergic neurons* are required. The technically demanding GAD stain proved difficult to perform *post hoc* after *in vitro* electrophysiological recordings. Thus, to determine the intrinsic membrane properties of GABAergic neurons controlling the sleep-wake cycle, our laboratory took advantage of *genetically modified mice expressing green fluorescent protein (GFP) under the control of the Gad1 (GAD67) promoter*. Several different types of mice expressing GFP or other fluorescent markers under the control of part or all of the GAD promoter are now available, but the best validated are the *GAD67-GFP knock-in mice* generated by Yuchio Yanagawa and colleagues (25). In our laboratory, we confirmed that the sleep-wake cycle and cortical rhythms are normal in these mice (26, 27) and that GFP selectively labels essentially all GABAergic neurons in the BF and brainstem (24, 28). GFP also delineates other known GABAergic neuronal groups controlling the sleep-wake cycle, e.g., the circadian pacemaker in the suprachiasmatic nucleus and histaminergic neurons in the tuberomammillary nucleus of the hypothalamus (Figure 1) (29). A great advantage of these mice is that *in vitro* recordings can target GABAergic neurons online, prior to recording. Furthermore, retrograde tracing studies of the projections of these neurons do not require the use of GAD staining (29). Other genetically modified mice expressing a red fluorescent marker (tdTomato) in PV neurons also proved useful in characterizing the properties of PV-containing, cortically projecting, BF GABAergic neurons (24, 30, 31).

While recording the activity of GABAergic neurons *in vivo* and determining their properties *in vitro* provided important clues to their function and potential ways to pharmacologically manipulate their activity, tests of their functional role require selective stimulation and inhibition experiments. The development of *mice expressing the bacterial enzyme Cre recombinase under the control of the GAD, vesicular GABA transporter (vGAT), or PV promoters* further advanced our knowledge concerning the role of GABAergic neurons. Use of these mice allowed selective *optogenetic* (33) and *designer receptor exclusively targeted by designer drugs (DREADD)* approaches (34). In these techniques, Cre recombinase-dependent (double-floxed), viral vector mediated transduction allows the introduction of excitatory and inhibitory channel/pumps, which can be activated by light (optogenetics) or modified G-protein coupled cholinergic receptors which are activated by a normally inert drug, clozapine-N-oxide (DREADD technique) into selected groups of neurons, i.e., GABAergic or PV neurons containing Cre Recombinase. Work currently underway using these approaches (35–37) has provided important evidence supporting a role of BF GABAergic/PV neurons in the promotion of wakefulness and cortical gamma rhythms.

In the following, we describe what is currently known about subcortical, ascending GABAergic *projection* neurons which are active during wakefulness (and rapid-eye-movement sleep) and regulate wakefulness, as well as the higher frequency rhythms typical of brain-activated states. We do not cover GABAergic neurons which are primarily sleep-active (10, 20, 38–40) or those involved in the descending control of muscle tone (41, 42). We also do not cover GABAergic *inter*neurons in the hippocampus and neocortex in detail, although they are extremely important in generating fast EEG rhythms (43–45) and are a target of ascending GABAergic projections (46–48). We begin in the brainstem and continue rostrally along the dorsal and ventral pathways of the ascending reticular activating system (ARAS), which include the thalamus and BF as their final nodes.

## Brainstem GABAergic Neurons Controlling Theta Rhythms

**Figure 2:**
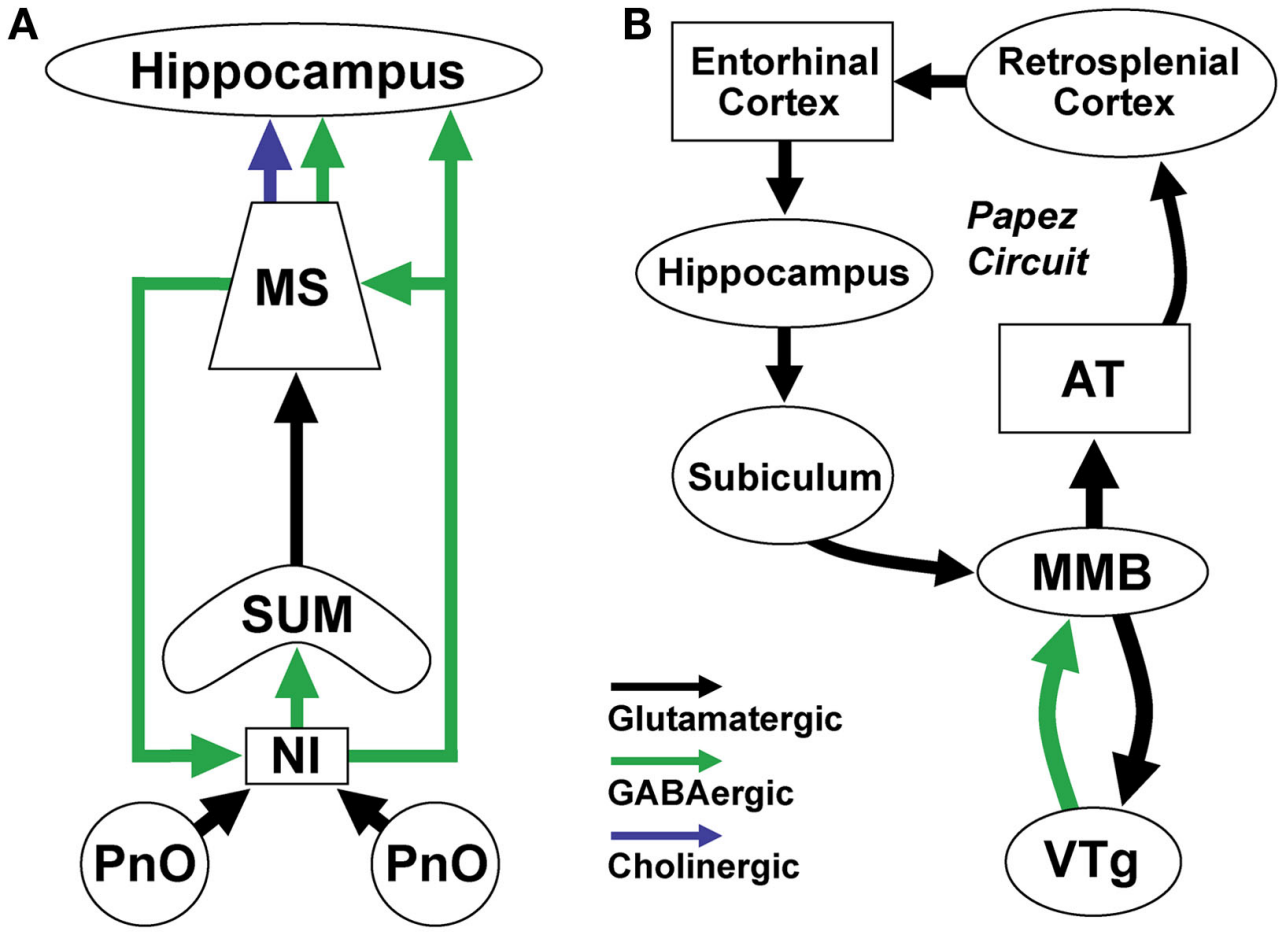
**Brainstem GABAergic projection neurons in the nucleus incertus (NI) and ventral tegmental nucleus of Gudden (VTg) regulate theta (4–8 Hz) rhythms important for spatial navigation and memory formation**. **(A)** NI GABAergic/relaxin-3 positive neurons receive input from neighboring pontine nucleus oralis (PnO) reticular neurons which increase their activity during active wakefulness and REM sleep. They project to and synchronize the activity of theta-rhythm related neurons in the supramammillary nucleus (SUM), medial septum (MS), and hippocampus. They receive return projections from a different population of septal GABAergic neurons. **(B)** VTg GABAergic projection neurons generate theta rhythmic activity through interactions with medial mammillary body glutamatergic neurons (MMB). MMB neurons transmit rhythmic theta-frequency activity to the anterior thalamus (AT) and through the rest of the Papez circuit. These two theta-generating circuits participate in synchronizing the activity of neurons involved in spatial navigation and memory by linking neurons representing information about the environment with those representing information about the position of the animal (51).

The brainstem has a relatively small number of ascending, wake/REM-active GABAergic neurons (49). However, a large number of locally projecting, GABAergic interneurons surround and control the activity of ascending brainstem cholinergic, monoaminergic, and glutamatergic systems involved in sleep-wake control (13, 14, 18, 28, 49). In this section, we focus on two brainstem nuclei with GABAergic projection neurons, the *nucleus incertus (NI)* and the *ventral tegmental nucleus of Gudden (VTg)* (Figures 1A,B), both of which are involved in control of theta (4–8 Hz) rhythms which dominate the cortical EEG in rodents during active wakefulness and REM sleep (50), and are important in synchronizing the activity of brain areas involved in spatial navigation and memory formation (Figure 2).

### Nucleus incertus

Several lines of evidence suggest that NI GABAergic neurons containing the neuropeptide relaxin-3 are involved in control of theta rhythms. Anatomical studies revealed that GABAergic, relaxin-3 positive neurons in the NI project to regions involved in theta rhythm generation such as the supramammillary nucleus, medial septum/diagonal band (MS/DB), and the hippocampus (52–55) (Figure 2A). Other projections target intralaminar thalamus, hypothalamus, and amygdala. Relaxin-3 positive fibers in the MS/DB contact the cholinergic and GABAergic/PV neurons projecting to the hippocampus, which are known to act as pacemakers for theta rhythm (56). Ultrastructural analysis indicates that relaxin-3 positive terminals form symmetrical contacts typical of GABAergic synapses (56). Conversely, NI receives return GABAergic projections from the septum and horizontal limb of the diagonal band (57) (Figure 2A).

The anatomical projections of NI neurons led to direct physiological tests of a role for NI in control of theta rhythm. Electrical stimulation of the NI in urethane-anesthetized rats induced theta rhythm in the hippocampus (58). Conversely, electrolytic lesions or pharmacological inhibition of the NI abolished theta rhythm evoked by stimulation of the neighboring pontine reticular formation (PnO) in urethane anesthetized animals (58).

How might NI neurons facilitate hippocampal theta rhythms? Initial single-unit recordings revealed that the majority of NI neurons did not fire rhythmically or fired rhythmically at frequencies (13–25 Hz) higher than theta rhythm frequencies (58), indicating that they are unlikely to be involved in converting tonic reticular input into phasic theta frequency firing. However, more recent findings suggest that there are two populations of NI neurons, a relaxin-3 positive subpopulation which is excited by corticotropin-releasing factor (CRF) and exhibits strong phase-locked firing with the ascending phase of hippocampal theta oscillations, and a relaxin 3-negative subpopulation which is unaffected by CRF and whose firing is not phase-locked with hippocampal theta (59). Taking together the anatomical and physiological findings, it seems plausible that the GABA/relaxin-3 NI neurons promote theta rhythm by synchronizing the firing of septohippocampal GABA/PV neurons. However, the precise roles of GABA and relaxin-3 in this process remain to be determined. Consistent with a role in the control of hippocampal theta rhythm, inactivation of NI with lidocaine impairs the acquisition and retrieval of spatial reference memory (60).

### VTg

Another ascending brainstem projection involved in theta generation involves the VTg region originally described by the German psychiatrist, Bernhard von Gudden (61). VTg consists of two small aggregations of GABAergic neurons located on either side of the midline between the dorsal and median raphe (28, 62–64) (Figure 1B). *In vivo* recordings in both urethane-anesthetized (65) and freely moving (66, 67) rats revealed that VTg neurons fire long-lasting bursts of action potentials at high-frequencies which are phase-locked with hippocampal theta rhythms. Our *in vitro* recordings from identified GABAergic VTg neurons in GAD67-GFP knock-in mice suggest that these long-lasting bursts are due to a low-threshold calcium spike and subsequent activation of a long-lasting calcium-activated sodium conductance (68, 69). Like other ascending GABAergic neurons, VTg neurons are very fast-firing *in vitro* (maximal firing rate >200 Hz) and have narrow action potentials with brief afterhyperpolarizations (68, 69). They also exhibit strong hyperpolarization-activated cation currents (H-currents), which are often present in rhythmically active neurons.

Unlike NI neurons, VTg neurons do not have projections to the structures most commonly linked to theta-rhythm generation, the medial septum, supramammillary nucleus, or hippocampus. Instead, the VTg shares strong reciprocal connections with glutamatergic neurons of the medial mammillary body (MMB, Figure 2B) (63, 70–72). A parallel GABAergic ascending projection system involved in transmitting head-direction information arises in the dorsal tegmental nucleus and projects to the lateral mammillary nucleus (73). As with the VTg, single-unit recording studies from the MMB have reported neurons which fire rhythmic bursts in synchrony with hippocampal theta rhythm (74, 75), although these bursts are shorter in duration and occur at a different phase with respect to hippocampal theta rhythms. Like VTg neurons, MMB neurons have low-threshold calcium spikes, which underlie the theta bursts recorded *in vivo* (68, 76).

The intrinsic membrane properties, neurotransmitter phenotype, and reciprocal connections of VTg and MMB neurons suggest a mechanism which may generate their theta activity (68): bursts of action potentials in glutamatergic MMB neurons lead to depolarization of VTg neurons and activation of low-threshold calcium channels; calcium influx into VTg neurons activates a calcium-activated cation conductance which prolongs the burst and increases the number of action potentials; long-lasting bursts in VTg neurons lead to a strong GABAergic inhibition of MMB neurons, de-inactivating the low-threshold calcium channels in MMB neurons, which are then activated once the VTg-mediated hyperpolarization subsides, restarting the cycle. Strong hyperpolarization-activated cation channels in VTg neurons help maintain the rhythmic firing by providing a depolarizing influence during the intraburst interval (68). Descending inputs to the MMB from the subiculum may act to synchronize theta rhythms in the hippocampus with those in the MMB-VTg circuit (74, 77, 78).

The major ascending output of the MMB innervates the medial and ventral parts of the anterior thalamus via axons which ascend within the mammillothalamic tract. Theta burst neurons have been recorded in the anterior thalamus, in particular in the ventral part targeted by the MMB (79, 80). The anterior thalamus in turn projects to the cingulate cortex and presubiculum, which is connected with the entorhinal cortex, one of the two main afferent inputs to the hippocampus. Thus, the VTg-MMB system may act as a theta rhythm generating system for the neural circuit described by Papez (51, 65, 79, 81, 82) (Figure 2B). Damage to the MMB→thalamus pathway in Korsakoff’s syndrome or via stroke results in diencephalic amnesia (73, 83). Similarly, neurotoxic lesions of the VTg in animals impair memory formation and learning (84).

### Summary

Two ascending GABAergic brainstem nuclei, the NI and VTg, play key roles in the generation of theta rhythms during waking and REM sleep (Figure 2). The NI promotes theta generation in the suprammillary-medial septal-hippocampal system involved in formation of spatial maps of the environment, whereas the VTg-MMB system promotes theta rhythms in the anterior thalamus head-direction system (85). Integration of information from these two systems allows an animal to represent its position within the environment, a key requirement for spatial and episodic memory formation/retrieval during waking and REM sleep (86).

## Ventral Tegmental Area GABAergic Neurons Involved in Arousal and Reward

In addition to the well-known dopaminergic neurons involved in reward and addiction, the midbrain ventral tegmental area (VTA) nucleus contains a substantial percentage (~20–35%) of GABAergic neurons (87–89). Important recent studies suggest that VTA GABAergic projection neurons, VTA GABAergic interneurons, and GABAergic inputs from the rostromedial tegmental nucleus (RMTg) are all involved in reward and reward-related arousal processes. VTA GABAergic projection neurons target widespread forebrain targets including the BF/preoptic area, amygdala, mediodorsal thalamus, and lateral hypothalamus, as well as arousal nuclei of the brainstem such as the dorsal raphe and deep mesencephalic nuclei (90). Sparser but functionally important projections target the prefrontal cortex (PFC) (87, 90, 91) and nucleus accumbens (NAcc) (90, 92, 93). A portion of the VTA GABAergic projection to PFC synapses onto neocortical GABAergic interneurons of unknown subtype (87), whereas the GABAergic VTA projection to NAcc preferentially targets cholinergic interneurons (94), suggesting that VTA GABAergic projection neurons indirectly modulate the activity of principal neurons in these structures.

### VTA GABAergic neurons are fast-firing and electrically coupled

*In vivo* extracellular recordings in halothane-anesthetized rats revealed that GABAergic VTA neurons exhibit a rapid (~19 Hz), cluster-type discharge pattern, short-duration action potentials, and lack of accommodation of firing during prolonged depolarizations (93). They were activated antidromically and orthodromically by stimulation of the internal capsule, confirming that they are projection neurons and were inhibited by electrical stimulation of the NAcc. Intracellular, sharp-electrode recordings *in vivo* revealed they had relatively depolarized resting membrane potentials (~−62 mV) and small action potentials (68 mV). *Post hoc* staining for neurobiotin and GABA confirmed that they were GABAergic and not dopaminergic (93). The expression of connexin-36 mRNA and protein, inhibition of prolonged discharges induced by high-frequency stimulation of the internal capsule by gap junction blockers, and the presence of neurobiotin dye coupling all suggest that VTA GABAergic neurons are electrically coupled, facilitating synchronization of their activity (95–97). *In vitro* recordings in GAD67-GFP knock-in mice indicated that VTA GABAergic neurons can be distinguished from dopamine neurons by their narrow action potentials and lack of an A-type potassium current (89).

### The discharge of VTA GABAergic neurons is associated with arousal

In unrestrained, unanesthetized rats, VTA GABAergic neurons discharged even move rapidly than in anesthetized animals (29 ± 6 Hz during active waking), and their discharge was markedly elevated during the onset of movement and prior to brain stimulation reward, suggesting a role in arousal/attention (98, 99). Firing rates during movement could reach as high as 100–200 Hz for 10–20 s (98). Consistent with the initial report (93), the discharge of VTA GABAergic neurons was strongly suppressed by deep chloral hydrate, ketamine, or halothane anesthesia, and was reduced during non-REM sleep (98). During REM-sleep, VTA GABAergic neurons increased their mean firing rate beyond that observed during active waking to ~52 Hz. Furthermore, during REM sleep enhanced firing was correlated with EEG gamma band activity. However, to date, a role for VTA GABAergic neurons in controlling particular EEG frequency bands has not been tested using selective excitation and inhibition experiments.

### VTA GABAergic neurons are excited by wake/arousal promoting neurotransmitters

Whole-cell recordings from two groups of non-dopaminergic, putative GABAergic VTA neurons demonstrated that they are strongly excited by the wake-promoting orexin/hypocretin peptides (100, 101). Putative VTA GABAergic neurons are also excited by other arousal/stress-related neuromodulators such as histamine, CCK, and substance P (101, 102). These studies did not distinguish effects on projection neurons from those on local GABAergic interneurons, an important future direction.

### Role of VTA GABAergic neurons in reward processes

**Figure 3:**
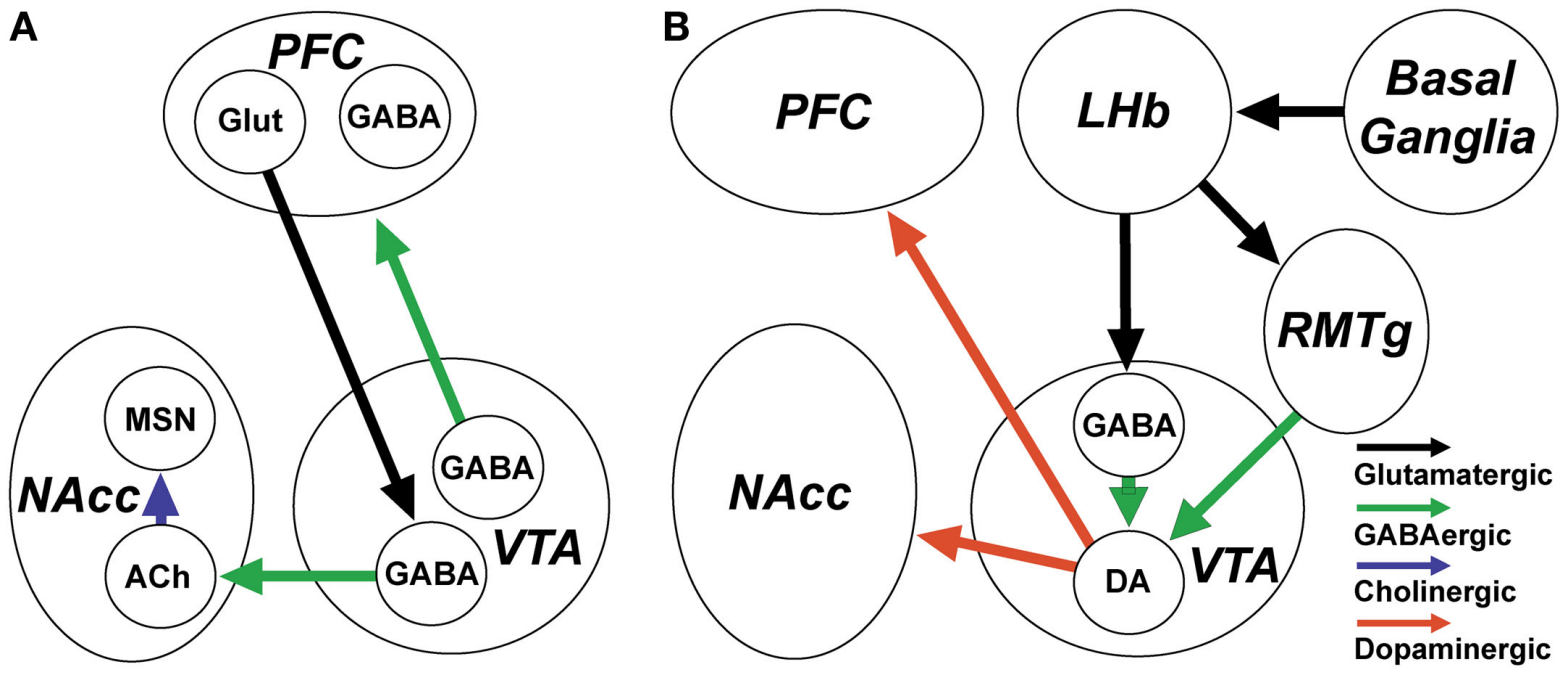
**GABAergic neurons and inputs to the ventral tegmental area (VTA) are involved in the control of reward and reward-related arousal**. **(A)** VTA GABAergic projection neurons innervate the prefrontal cortex (PFC) and nucleus accumbens (NAcc). They increase their discharge in association with arousal and in anticipation of reward. VTA neurons projecting to PFC target both principal neurons and cortical GABAergic interneurons. VTA GABAergic neurons projecting to the NAcc receive input from PFC and specifically target NAcc cholinergic interneurons, which regulate plasticity of medium spiny neurons (MSN). **(B)** The activity of dopaminergic VTA neurons which encode unexpected rewards and project to NAcc and PFC is under the control of local GABAergic interneurons and GABAergic inputs from the rostromedial tegmental nucleus (RMTg). RMTg and local VTA GABAergic neurons are excited by lateral habenula (LHb) glutamatergic neurons which encode expected rewards or the absence of rewards, based on inputs from the basal ganglia.

Recent work has elaborated on the role of VTA GABAergic neurons in reward-related processes (Figure 3A). This work suggests that motivationally important information from the PFC is conveyed to VTA GABAergic neurons which target NAcc. Anatomical studies showed that PFC projections to VTA selectively target GABAergic neurons which project to the NAcc, but not those projecting back to the PFC (103). *In vivo* recordings demonstrated a task-dependent increase in coherence at gamma band frequencies between PFC and VTA during a working memory task, likely reflecting transmission of information from PFC to fast-firing VTA GABAergic neurons (104). The projection of VTA GABAergic neurons to the NAcc selectively targets and inhibits cholinergic NAcc interneurons (94). Activation of the VTA GABA→NAcc pathway caused a pause in the discharge of cholinergic interneurons, resembling that previously observed in animals learning stimulus-outcome associations. Furthermore, optogenetic activation of this pathway enhanced discrimination of a motivationally important stimulus paired with an aversive outcome (94).

### VTA GABAergic interneurons control dopaminergic activity

Many drugs of abuse, such as opiates (105) and benzodiazepines (106), inhibit VTA GABAergic interneurons, leading to increased dopamine release via disinhibition. Conversely, optogenetic activation of VTA GABAergic neurons *in vivo* disrupts reward consummatory behavior (107). VTA GABAergic neurons, likely interneurons, identified using optogenetic activation, inhibited neighboring dopaminergic neurons, and encoded the presence of expected rewards (108). Disruption of this coding by drugs of abuse likely contributes to mechanisms of addiction.

### The GABAergic neurons of the RMTg convey aversive information to VTA dopaminergic neurons

The posterior part of the VTA and its extension into the tegmental region of the reticular formation (collectively known as the posterior VTA or RMTg nucleus) contains a population of ascending GABAergic neurons which project to and regulate the activity of more rostrally located VTA dopaminergic neurons (109, 110). Unlike VTA GABAergic neurons, however, RMTg neurons do not have substantial projections to the forebrain (109). Recent studies implicate these GABAergic neurons in aversive behavior and reward prediction through a basal ganglia→lateral habenula→RMTg circuit (111, 112) (Figure 3B). Lateral habenula neurons are excited by stimuli that indicate the absence of a reward (113), through an excitatory pathway arising in the basal ganglia (112), leading to excitation of RMTg GABAergic neurons and subsequent inhibition of reward-related midbrain dopaminergic neurons and pedunculopontine neurons (111, 114). Like VTA GABAergic interneurons, RMTg neurons are inhibited by μ-opioids and cannabinoids (115). Further experiments suggested that the RMTg may be the most important site for disinhibitory effects of these compounds on dopaminergic activity (116).

### Summary

Ventral tegmental area GABAergic projection neurons promote reward and reward-related arousal through their mesoaccumbens and mesocortical projections, which notably target interneurons in both structures. Conversely, VTA GABAergic interneurons and RMTg GABAergic inputs, encoding expected reward or the absence of rewarding stimuli, exert a tonic inhibitory effect on VTA dopaminergic neurons (Figure 3).

## Ascending Hypothalamic GABAergic Systems Controlling the Thalamus and Neocortex

Retrograde tracing studies identified four major hypothalamus systems projecting to the cerebral cortex (117). Of these four systems, three subsets of neurons in the tuberal part of the lateral hypothalamic contain GABA: GABAergic neurons in the ventral part of the zona incerta (ZI), melanin concentrating hormone (MCH) neurons, and tuberomammillary histaminergic neurons. MCH neurons are sleep-active and sleep-promoting and are therefore not considered further in this section. Although histaminergic neurons promote arousal (118) and they contain the biosynthetic machinery for GABAergic transmission (119), the functional role is poorly understood (120). Therefore, we concentrate in this section on ZI GABAergic projection neurons.

Glutamic acid decarboxylase immunostaining coupled with retrograde tracing confirmed the presence of a group of GABAergic projection neurons in the ZI (121). Many of these neurons may also contain the neuropeptide α-melanocyte stimulating hormone (122, 123), or a closely related peptide (124). GABAergic ZI neurons project heavily to the thalamus (125) as well as to the neocortex (121). Immunostaining also revealed a population of PV-containing neurons in this same area, suggesting that as in other areas, these GABAergic neurons are likely to be fast-firing. *In vitro* recordings from the ventral ZI region revealed that most neurons discharged spontaneously at high rates (9.3 Hz median firing rate) and also exhibited rhythmic firing (126). In the same study, *in vivo* recordings in urethane-anesthetized animals revealed firing at 3–4 Hz. However, in a different study under light ketamine or urethane anesthesia, ventral ZI neurons discharged much more rapidly, with mean rate of 26 Hz (127). The role of ZI projections to the cortex has still not been explored in any detail, but recent studies suggest an important regulation of higher order sensory nuclei of the thalamus (see next section).

## GABAergic Neurons Controlling Thalamocortical Activity during Wakefulness

The midline and intralaminar thalamic nuclei represent the final node of the dorsal portion of the reticular activating system (9). These nuclei, and the primary thalamic sensory relay nuclei, are under strong inhibitory control from GABAergic neurons in several different regions. The most prominent, widespread, and well-known GABAergic input arises from the thalamic reticular nucleus (TRN). A more restricted input to the midline centromedian-parafascicular nucleus (CM-Pf) and motor thalamus (ventrolateral and ventromedial nuclei) originates from the output of the basal ganglia, the globus pallidus, pars interna (GPi) and the substantia nigra, pars reticulata (SNr) (128–130). Other GABAergic inputs to the midline thalamus arise in the ZI (125) and BF (131). In general, all of these GABAergic inputs maintain a high rate of tonic inhibition in the thalamic relay nuclei, which is likely important in suppressing unimportant information and unnecessary motor activity. However, in situations requiring high attention and responses to important situations, this tonic input is transiently suppressed allowing enhanced arousal/attention (disinhibition of higher-order nuclei) and sensorimotor transmission (disinhibition of first-order sensory and motor nuclei).

### Thalamic reticular nucleus

In addition to being crucially involved in generating sleep spindles during non-REM sleep (132, 133), TRN neurons maintain a high rate of tonic firing during wakefulness (134–136), which serves to prevent unimportant sensory information being transmitted through the thalamic relay nuclei to the cortex. TRN neurons receive excitatory inputs from noradrenergic and serotonergic neurons which maintain this high firing rate during wakefulness (137). Reduced activity of sensory-related TRN neurons occurs prior to correct performance in attention tasks (138), leading to disinhibition of relay neurons (139). The origin of this reduced activity of TRN neurons is unclear. TRN receives inhibitory GABAergic/PV projections from globus pallidus (140), substantia nigra pars reticulata (141), and BF GABAergic/PV neurons (142, 143). Input from BF GABAergic/PV neurons is particularly interesting, considering the strong inputs to these BF neurons from PFC regions involved in processing novelty (144–146) and their strongly state-dependent firing (see next section). Other experiments have suggested that TRN may be involved in gamma-band oscillations through entrainment of the activity of thalamocortical neurons (147–149).

### Basal ganglia GABAergic input to the thalamus

The two main GABAergic output nuclei of the basal ganglia are the GPi and SNr. GPi and SNr project to thalamic motor output nuclei and to the centromedian-parafascicular nucleus (CM-Pf), which is one of the “non-specific” thalamic nuclei regulating the level of arousal through widespread, diffuse projections to the cortex (150). CM-Pf also has a prominent projection to the striatum, enhancing activity in cortico-basal ganglia-thalamocortical circuits. Electrical or pharmacological activation of CM-Pf in rodents enhances behavioral arousal and recovery from anesthesia, supporting a role in control of consciousness (151–154). Furthermore, changes in CM-Pf activity precede loss of consciousness caused by anesthetics or transitions into sleep (155). Thus, GABAergic control of CM-Pf by the basal ganglia output nuclei is likely to be extremely important in control of arousal and consciousness.

Neurons in GPi and SNr express very high levels of the α1 subunit of the GABA_A_ receptor, the target of the hypnotic, zolpidem (156–159) in rodents and in humans (160). Interestingly, in brain damaged patients, it has been hypothesized that the activity of these neurons is pathologically enhanced, resulting in an inhibition of thalamic and pedunculopontine neurons and loss of consciousness (161). Thus, suppression of their activity by zolpidem may result in a paradoxical arousing effect in some patients (162–166).

### ZI GABAergic input to the thalamus

In addition to direct projections to the cortex, ZI GABAergic projection neurons innervate the thalamus, superior colliculus, and brainstem (125, 167). Thalamic projections of ZI GABAergic neurons are particularly strong to higher-order nuclei, where they effect a tonic inhibition of sensory transmission (125, 167). In the rodent, ZI neurons receive a strong input from trigeminal axons transmitting sensory information from the whiskers, leading to excitation of ZI GABAergic neurons and preventing excitation of the posterior thalamic group neurons (127). This feed-forward inhibition can be overcome by excitation of the motor cortex and activation of an intra-incertal GABAergic circuit (168). Many other cortical areas also converge on ZI GABAergic neurons, which through their widespread cortical, thalamic, and brainstem projections are in a position to globally modulate brain arousal (169). Cholinergic stimulation *in vitro* or *in vivo* in anesthetized rats inhibited the firing of ventral ZI neurons, suggesting that high arousal states involving increased acetylcholine release lead to suppression of ZI neuronal firing and promotion of sensory transmission (126).

### Summary

GABAergic projection neurons in the TRN, GPi, SNr, and ZI act to tonically inhibit various thalamic relay neurons during wakefulness. Suppression of their firing by cortical or subcortical inputs is a powerful mechanism to increase thalamic activity and thereby increase attention, arousal, sensory processing, and motor activity in a context-dependent manner.

## Cortically Projecting BF GABAergic Neurons Promote Cortical Activation and Wakefulness

The BF represents the final node of the ventral portion of the brainstem ARAS. Rostral BF neurons in the medial septum and vertical limb of the diagonal band project to the hippocampal formation, whereas intermediate and caudally located BF neurons in the horizontal limb of the diagonal band, magnocellular preoptic area, ventral pallidum, substantia innominata, and nucleus basalis project to the neocortex, as well as to the thalamus, lateral hypothalamus, and brainstem (11, 12, 16, 46, 47, 170). Many studies have focused on the arousing effects of cortically projecting cholinergic neurons in this region (171), which are among the first to degenerate in Alzheimer’s disease (172). However, recent studies suggest that neighboring GABAergic projection neurons and interneurons may be equally important in cortical activation. Juxtacellular labeling experiments *in vivo* identified a significant minority of BF GABAergic neurons, which are fast-firing (20–60 Hz) and increase their firing rate during wakefulness and REM sleep (19). Recent preliminary experiments showed that pharmacogenetic (DREADD) stimulation of BF GABAergic neurons strongly enhances wakefulness (37). Similarly, initial experiments suggest that optogenetic stimulation of BF PV neurons is wake-promoting (35), in addition to playing a role in control of cortical gamma oscillations (36).

### Anatomy and projections of BF GABAergic neurons

Anterograde tracing studies coupled with staining for GAD showed that cortically projecting GABAergic neurons make up approximately one-third of the BF projection to the cortex. Importantly, BF GABAergic projections to the neocortex and hippocampus prominently target interneurons containing PV and somatostatin, which are thought to be involved in gamma and theta oscillations (46–48). Caudal/intermediate BF GABAergic neurons projecting to the neocortex, as well as more rostrally located BF GABAergic neurons projecting to the hippocampus, preferentially target GABAergic interneurons (46, 47, 173), in particular PV-containing fast-spiking interneurons (174) involved in theta (175) and gamma oscillations (44, 45). Somatostatin-containing cortical interneurons which target the dendrites of pyramidal neurons and appear tuned for theta-frequency firing are also a target of BF cortically projecting GABAergic neurons, as are cortical interneurons containing calbindin (173). BF PV and GABAergic neurons also project to the TRN (142, 143) and less prominently to the mediodorsal nucleus (131, 170). At present, it is unclear if only BF PV/GABAergic neurons or also other types of BF non-PV/GABAergic neurons project to TRN. The function of the BF projection to TRN is currently unresolved but this projection may be involved in attentional suppression of TRN discharge (see previous section) and/or suppression of spindle-related bursting in TRN during wakefulness and REM sleep.

### Subtypes of cortically projecting BF GABAergic neurons

**Figure 4:**
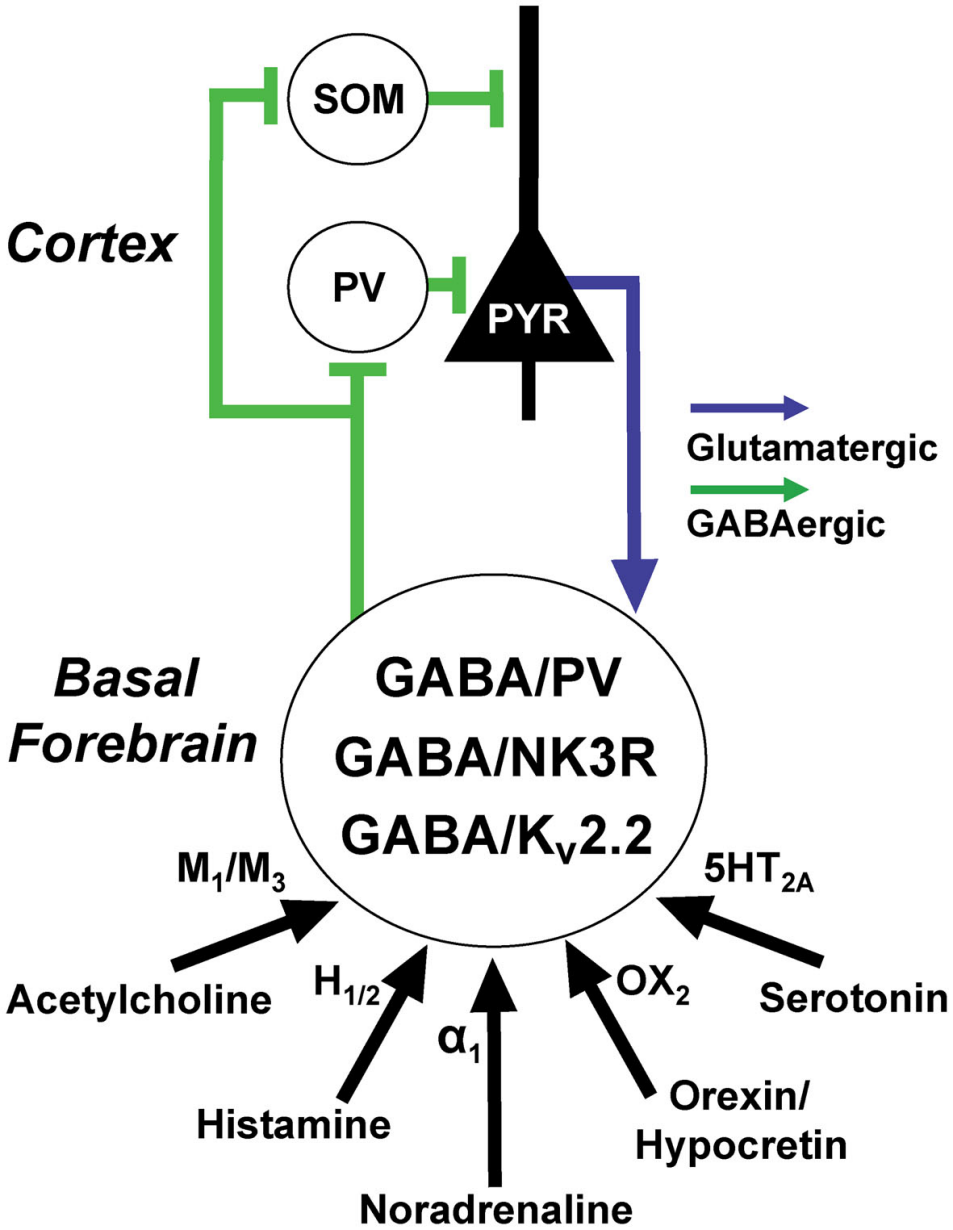
**Basal forebrain (BF) GABAergic neurons are excited by wake promoting neuromodulators and promote gamma rhythms in the cortex via projections to cortical GABAergic interneurons**. At least three, largely separate, populations of BF GABAergic neurons express the calcium-binding protein, parvalbumin (PV), the neurokinin-3 receptor (NK3R), and the potassium channel Kv2.2. BF GABAergic neurons can also be subdivided according to the amplitude and kinetics of their hyperpolarization-activated cation currents (H-currents). GABA/PV neurons in caudal/intermediate parts of the BF appear to be important in regulating cortical gamma oscillations through their synchronization of cortical PV interneurons. Rostral BF GABA/PV neurons (not shown) regulate hippocampal theta and gamma oscillations. The functions of the NK3R and Kv2.2. subpopulations are less well-understood but they also appear to be wake-promoting. Cortical and hippocampal projections of identified BF GABAergic or PV fibers preferentially appose GABAergic interneurons, including fast-spiking, somatic targeting PV interneurons, and dendrite-targeting somatostatin (SOM) interneurons. Return projections from the cortex target cortically projecting BF PV neurons and possibly other GABAergic subpopulations but avoid cholinergic neurons.

Several subtypes of cortically projecting GABAergic neurons have been identified in the BF based on the expression of different neurochemical markers (Figure 4). The most well-known are neurons containing the calcium-binding protein PV. Other, largely separate subsets express the neurokinin-3 receptor (176) and the potassium channel Kv2.2 (177, 178). *In vitro* recordings suggest that large-sized, cortically projecting GABAergic and PV neurons can be subdivided into two groups based on the amplitude and kinetics of their H-current (24).

### PV neurons: Anatomy and in vivo recordings

The vast majority of PV-containing neurons in the BF are GABAergic (16, 24). PV is a marker for cortically projecting BF GABAergic neurons (16). In the rat, immunohistochemical staining for PV labeled approximately 90% of GAD-stained neurons, retrogradely labeled from the orbitofrontal and somatosensory cortex (16). In the GAD67-GFP knock-in mouse, PV was observed in ~25% of large (>20 μm) diameter, putative cortically projecting GABAergic neurons, whereas, overall PV was present in 6.7% of all BF GABAergic neurons (24). PV-containing GABAergic neurons in the rostral part of the BF (medial septum and vertical limb of the diagonal band) project to the hippocampus, and are critically involved in control of hippocampal theta rhythm (179, 180), and associated hippocampal gamma rhythms, whereas PV-containing neurons in intermediate and caudal BF regions project to the neocortex and regulate neocortical gamma oscillations (35). Two populations of medial septal PV neurons discharge at opposing phases of the hippocampal theta rhythm. *Post hoc* identified PV neurons recorded in anesthetized rats discharged rapidly in bursts or clusters in association with cortical activation induced by tail pinch (17). In unanesthetized mice, two optogenetically identified PV neurons discharged irregularly in the gamma range (20–60 Hz) during wakefulness and REM sleep (36). Transduction of BF PV fibers with channelrhodopsin2-enhanced yellow fluorescent protein fusion proteins revealed BF PV neurons appose cortical PV interneurons, consistent with a role in control of cortical gamma oscillations (36). Direct tests of this hypothesis using optogenetic techniques demonstrated that both rhythmic and non-rhythmic stimulation of BF PV neurons preferentially enhanced cortical EEG power at gamma frequencies, whereas optogenetic inhibition of BF PV neurons reduced the ability of the cortex to respond at 40 Hz in response to a train of auditory stimuli delivered at 40 Hz (36). Together, these data strongly implicate BF PV neurons in the behavioral state-related increases in cortical gamma oscillations, which are observed during wakefulness and REM sleep (181, 182).

### PV neurons: In vitro recordings and intrinsic properties

*In vitro* recordings from identified PV neurons using genetically modified mice expressing fluorescent markers, *post hoc* staining (183), or based on intrinsic membrane properties have revealed many similarities between MS/DB GABAergic/PV neurons projecting to the hippocampus and those projecting to the neocortex. Both groups are very fast-firing (24, 183), likely due to their expression of the delayed rectifier Kv3.1 (*Kcnc1*) potassium channel (184), and whose extremely fast kinetics enables fast repolarization of the action potential (185). Both groups of BF GABAergic/PV neurons exhibit a “depolarizing sag” during hyperpolarizing current pulses due to a hyperpolarization-activated cation current (H-current) (24, 183), a property which distinguishes them from cortical PV interneurons and TRN GABAergic/PV neurons. This current counteracts prolonged hyperpolarization and is also often present in neurons which show rhythmic firing, providing a depolarizing influence in the interburst interval following afterhyperpolarizations. Thus, this current may be important for the cluster and burst-like firing of these neurons recorded *in vivo* (17). In fact, infusion of an H-current blocker into the MS/DB impairs theta rhythm generation (186, 187). Like PV cortical interneurons, BF GABAergic and PV neurons showed evidence of electrical coupling (24).

### Neurokinin3 receptor immunoreactive neurons

The Neurokinin3 Receptor (NK3R) is the most selective receptor for neurokinin B, produced from the precursor preprotachykinin (PPTB). PPTB is present in a small subset (5%) of projection neurons in the neostriatum which projects to the substantia innominata (188). Neurokinin B and NK3R are essential for normal reproduction (189). Thus, one plausible functional role for this population of BF GABAergic neurons is control of sexual arousal. Immunohistochemical staining in rats and in GAD67-GFP knock-in mice revealed that NK3R is present on a distinct subset of cortically projecting BF GABAergic neurons (176). About 92% of NK3 receptor positive neurons showed signals for GAD67 mRNA (176). Only 10–15% of NK3R expressing neurons were PV-positive and only 1.7% of PV-positive neurons were NK3R positive (176). About 1.7% of NK3R neurons were positive for calretinin and none contained calbindin, NPY, or somatostatin.

### Kv2.2 channel immunoreactive neurons

The potassium channel Kv2.2 is abundantly expressed in one group of BF GABAergic neurons (177). Less than 4% of Kv2.2 immunoreactive neurons stained positively for PV, establishing this as a separate group of GABAergic neurons (178). Sleep deprivation experiments showed that this group of neurons expressed more Fos, suggesting that this is a wake-active group of GABAergic neurons. Furthermore, knockout of the Kv2.2 channel led to more activity and promotion of wakefulness over sleep (178).

### Neurotransmitter regulation of wake-promoting BF GABAergic neurons

Our recent *in vitro* recordings in GAD67-GFP knock-in mice and PV-Tomato mice revealed that putative cortically projecting BF GABAergic and PV neurons are strongly excited by cholinergic inputs (31). Similarly, MS/DB GABAergic/PV neurons projecting to the hippocampus are excited by cholinergic agonists (190). Bath application of cholinergic agonists revealed that septohippocampal neurons are excited via M_3_ receptors (191) and indirectly by activation of nicotinic receptors on glutamatergic neurons (192). Two subpopulations of putative neocortically projecting BF GABAergic neurons (large Ih, small Ih) are excited via M_1_ muscarinic receptors and M_3_ muscarinic receptors, respectively (31). Optogenetic stimulation of cholinergic fiber terminals revealed an additional excitatory effect mediated by nicotinic receptors (31). Interestingly, blockade of cholinergic receptors in the BF blocked the ability of optogenetic stimulation of cholinergic neurons to increase wakefulness, indicating that cholinergic modulation of behavioral state may depend on local interactions with BF GABAergic and/or glutamatergic neurons (193). Septohippocampal GABAergic/PV neurons are also excited by noradrenaline (194), histamine (195), serotonin (196), and orexin/hypocretins (197, 198). Preliminary experiments suggest that this is also true for putative neocortically projecting GABAergic neurons (32). Anatomical tracing studies revealed that PV containing BF neurons receive direct input from cortex (144) and dopaminergic inputs from the substantia nigra-VTA (199).

### Summary

Several subsets of wake/REM active GABAergic neurons are present in the BF (Figure 4). They are strongly excited by wake-promoting neuromodulatory systems. Their projections target interneurons in the hippocampus and neocortex, as well as TRN GABAergic/PV neurons allowing control of cortical rhythms, attention, and wakefulness. The precise functional role of these different subtypes awaits further study but BF PV projection neurons appear to have a particular role in control of cortical gamma oscillations.

## Wake-Active GABAergic Neurons are Fast-Firing, Whereas Sleep-Active GABAergic Neurons are Slow Firing

*In vitro*, most putative ascending subcortical GABAergic neurons are fast-firing neurons, both in terms of their spontaneous firing rate and their maximum firing frequency. Those which contain PV are even faster-firing and have extremely brief action potentials and afterhyperpolarizations (24). Similarly, *in vivo*, these subpopulations of GABAergic neurons show high rates of firing during waking and REM sleep. This fast-firing is at least partly due to the presence of Kv3.1 and Kv3.3 potassium channels. These channels are present in cortical interneurons, TRN neurons, and BF GABAergic neurons projecting to the hippocampus and neocortex. Knockout of Kv3.1 channels alone leads to a relatively mild arousal phenotype involving fourfold increased gamma (20–60 Hz) activity during wakefulness and reduced delta oscillations during all states (200). Given that GABAergic/PV neurons are thought to be involved in promoting gamma activity, these results suggest that developmental compensation occurs. Knockout of the Kv3.3 channel led to no discernible phenotype. However, double knockout mice lacking the genes encoding both of these proteins have severe deficits in sleep-wake behavior (201–203).

In contrast to the fast-firing of wake/REM sleep active GABAergic neurons, the majority of sleep-active, presumptive GABAergic neurons in the ventrolateral preoptic area, median preoptic area, and lateral hypothalamic area (MCH neurons) tend to be silent at rest and fire slowly (<15 Hz), even during sleep, the state when they discharge fastest (20, 38, 40, 204–206). These differences in firing rates suggest alternative complements of voltage-gated ion channels and neurotransmitter receptors in wake and sleep-active GABA neurons. Thus, differential pharmacological modulation may be possible.

## Wake-Active GABAergic Neurons may be a Prominent Target of Hypnotic Agents

As described in the previous section, most wake-promoting GABAergic neurons are neurons which exhibit a high spontaneous and maximal firing rate, consistent with a role in controlling the fast theta, beta, and gamma-band EEG oscillations typical of wakefulness. GABAergic hypnotic agents such as diazepam, zolpidem (Ambien^TM^, α1 subunit selective agent), and eszopiclone (Lunesta^TM^, acts at both α3 and α1-containing GABA_A_ receptors), and anesthetic agents such as propofol are often considered to act by enhancing the inhibitory action of sleep-active preoptic GABAergic neurons on aminergic and cholinergic neuromodulatory systems. However, lesions or inactivation of these neuromodulatory systems have little effect on total amounts of sleep and waking [reviewed in Ref. (9)], suggesting that this explanation may not be correct. Furthermore, genetic removal of GABA_A_ receptors from histamine neurons did not alter sleep-wake behavior and did not alter the loss of righting reflex usually produced by propofol administration (207). Perhaps, more likely is that these agents act by inhibiting the discharge of ascending GABAergic neurons and slowing the inhibitory postsynaptic potentials in their target neurons, disrupting their entrainment of cortical and thalamocortical neurons in the fast frequency bands normally observed during wakefulness. α1 subunits, considered the main mediator of sedative actions of benzodiazepines and the target of zolpidem (Ambien), are expressed at very high levels by GABAergic projection neurons in GPi and SNr (157), VTA (106, 208), and by BF PV neurons (209). Examination of the Allen mouse brain atlas also suggests very high levels of GABAergic α1 subunits in the VTg and NI. α3-subunits are expressed at high levels in TRN GABAergic neurons and in BF PV neurons. Thus, a major mechanism of action of hypnotics/sedatives acting at these receptors may be inhibition of ascending subcortical GABAergic neurons which promote high-frequency EEG rhythms, facilitating the slower EEG rhythms typical of non-REM sleep.

## Summary and Conclusion

Recent technological advances have led to the identification and characterization of the properties of several groups of ascending subcortical GABAergic neurons, which are active during wakefulness and REM sleep, and may play a role in the generation/maintenance of these states and/or the high-frequency EEG oscillations with which they are associated. In particular, optogenetic and pharmacogenetic techniques exhibit great promise in discerning the functional role of these neurons since they allow selective neuronal excitation and inhibition experiments to be performed. Optogenetic tools are particularly useful for dissecting out their role in EEG rhythms due to their fast temporal resolution (34, 35). Conversely, pharmocogenetic tools may be particularly useful for elucidating their role in behavioral state control due to their long duration of action (10, 210).

Two groups of GABAergic neurons in the brainstem NI and VTg nuclei play important roles in the control of theta rhythms involved in spatial navigation and memory processes through their projections to the medial septum, supramammillary and medial mammillary nuclei. Another group of brainstem/midbrain GABAergic neurons in the RMTg region regulates the activity of VTA dopamine neurons involved in reward processes. GABAergic neurons in the VTA itself project to the cortex and NAcc and act to modulate attentive processes associated with reward. Several groups of GABAergic neurons in the thalamic reticular nucleus, ZI, and basal ganglia output nuclei control the activity of thalamic relay nuclei, suppressing unimportant sensory information and unnecessary motor activity. GABAergic neurons in the caudal/intermediate BF and medial septum/diagonal band project to neocortical and hippocampal GABAergic interneurons, and control theta and gamma oscillations. BF GABAergic/PV neurons in particular play an important role in control of neocortical gamma oscillations.

How do ascending subcortical GABAergic systems turn a negative (inhibitory postsynaptic effects) into a positive (cortical activation/arousal)? Ascending cortically projecting GABAergic neurons in the BF, VTA, and possibly also the ZI, target inhibitory neocortical interneurons, allowing disinhibitory effects and entrainment of fast cortical oscillations. Thalamic-targeting GABAergic neurons exert tonic inhibitory control, which can be suppressed in behaviorally important situations, potentiating thalamocortical transmission. Ascending GABAergic neurons exhibit narrow action potentials, brief hyperpolarizations, and are fast-firing. They often exhibit burst or cluster firing, and like cortical interneurons many are electrically coupled, properties which will enhance their action on their post-synaptic targets and enable them to synchronize their activity into fast oscillations. Most ascending, wake-active GABAergic neurons also express potassium channels and GABA_A_ receptor subunits, which are the targets of anticonvulsant and hypnotic/anesthetic agents. Thus, further study of these neurons to determine their functional role and neuropharmacology is likely to be very important in order to develop novel therapeutic compounds to modulate cortical activation, memory, reward, and sleep. Furthermore, understanding the properties of ascending GABAergic neurons may allow novel treatments for diseases involving disorders of cortical activation and wakefulness.

## Conflict of Interest Statement

The authors declare that the research was conducted in the absence of any commercial or financial relationships that could be construed as a potential conflict of interest.

## References

[1] Valenstein ES The War of the Soups and the Sparks. New York, NY: Columbia University Press (2005).

[2] Jouvet M Biogenic amines and the states of sleep. Science (1969) 163:32–41. 10.1126/science.163.3862.32 4303225

[3] Hobson JA McCarley RW Wyzinski PW. Sleep cycle oscillation: reciprocal discharge by two brainstem neuronal groups. Science (1975) 189:55–8. 10.1126/science.1094539 1094539

[4] McCarley RW Hobson JA. Neuronal excitability modulation over the sleep cycle: a structural and mathematical model. Science (1975) 189:58–60. 10.1126/science.1135627 1135627

[5] Winsky-Sommerer R. Role of GABAA receptors in the physiology and pharmacology of sleep. Eur J Neurosci (2009) 29:1779–94. 10.1111/j.1460-9568.2009.06716.x 19473233

[6] Sherin JE Shiromani PJ McCarley RW Saper CB. Activation of ventrolateral preoptic neurons during sleep. Science (1996) 271:216–9. 10.1126/science.271.5246.216 8539624

[7] Lu J Greco MA Shiromani P Saper CB. Effect of lesions of the ventrolateral preoptic nucleus on NREM and REM sleep. J Neurosci (2000) 20(10):3830–42. 10804223 10.1523/JNEUROSCI.20-10-03830.2000 PMC6772663

[8] Szymusiak R Gvilia I McGinty D Hypothalamic control of sleep. Sleep Med (2007) 8:291–301. 10.1016/j.sleep.2007.03.013 17468047

[9] Brown RE Basheer R McKenna JT Strecker RE McCarley RW Control of sleep and wakefulness. Physiol Rev (2012) 92:1087–187. 10.1152/physrev.00032.2011 22811426 PMC3621793

[10] Anaclet C Ferrari L Arrigoni E Bass CE Saper CB Lu J The GABAergic parafacial zone is a medullary slow wave sleep-promoting center. Nat Neurosci (2014) 17(12):1217–24. 10.1038/nn.3789 25129078 PMC4214681

[11] Gritti I Mainville L Jones BE. Codistribution of GABA- with acetylcholine-synthesizing neurons in the basal forebrain of the rat. J Comp Neurol (1993) 329:438–57. 10.1002/cne.903290403 8454735

[12] Gritti I Mainville L Mancia M Jones BE. GABAergic and other noncholinergic basal forebrain neurons, together with cholinergic neurons, project to the mesocortex and isocortex in the rat. J Comp Neurol (1997) 383:163–77. 10.1002/(SICI)1096-9861(19970630)383:2<163::AID-CNE4>3.3.CO;2-T 9182846

[13] Maloney KJ Mainville L Jones BE. Differential c-Fos expression in cholinergic, monoaminergic, and GABAergic cell groups of the pontomesencephalic tegmentum after paradoxical sleep deprivation and recovery. J Neurosci (1999) 19:3057–72. 10191323 10.1523/JNEUROSCI.19-08-03057.1999 PMC6782283

[14] Maloney KJ Mainville L Jones BE. c-Fos expression in GABAergic, serotonergic, and other neurons of the pontomedullary reticular formation and raphe after paradoxical sleep deprivation and recovery. J Neurosci (2000) 20(12):4669–79. 10844036 10.1523/JNEUROSCI.20-12-04669.2000 PMC6772475

[15] Maloney KJ Mainville L Jones BE. c-Fos expression in dopaminergic and GABAergic neurons of the ventral mesencephalic tegmentum after paradoxical sleep deprivation and recovery. Eur J Neurosci (2002) 15:774–8. 10.1046/j.1460-9568.2002.01907.x 11886456

[16] Gritti I Manns ID Mainville L Jones BE. Parvalbumin, calbindin, or calretinin in cortically projecting and GABAergic, cholinergic, or glutamatergic basal forebrain neurons of the rat. J Comp Neurol (2003) 458:11–31. 10.1002/cne.10505 12577320

[17] Duque A Balatoni B Detari L Zaborszky L. EEG correlation of the discharge properties of identified neurons in the basal forebrain. J Neurophysiol (2000) 84(3):1627–35. 10980032 10.1152/jn.2000.84.3.1627

[18] Boucetta S Jones BE. Activity profiles of cholinergic and intermingled GABAergic and putative glutamatergic neurons in the pontomesencephalic tegmentum of urethane-anesthetized rats. J Neurosci (2009) 29:4664–74. 10.1523/JNEUROSCI.5502-08.2009 19357291 PMC6665745

[19] Hassani OK Lee MG Henny P Jones BE. Discharge profiles of identified GABAergic in comparison to cholinergic and putative glutamatergic basal forebrain neurons across the sleep-wake cycle. J Neurosci (2009) 29:11828–40. 10.1523/JNEUROSCI.1259-09.2009 19776269 PMC2790860

[20] Hassani OK Henny P Lee MG Jones BE. GABAergic neurons intermingled with orexin and MCH neurons in the lateral hypothalamus discharge maximally during sleep. Eur J Neurosci (2010) 32:448–57. 10.1111/j.1460-9568.2010.07295.x 20597977 PMC2921479

[21] Celio MR Heizmann CW Calcium-binding protein parvalbumin as a neuronal marker. Nature (1981) 293:300–2. 10.1038/293300a0 7278987

[22] Celio MR. Parvalbumin in most gamma-aminobutyric acid-containing neurons of the rat cerebral cortex. Science (1986) 231:995–7. 10.1126/science.3945815 3945815

[23] Kawaguchi Y Kondo S. Parvalbumin, somatostatin and cholecystokinin as chemical markers for specific GABAergic interneuron types in the rat frontal cortex. J Neurocytol (2002) 31:277–87. 10.1023/A:1024126110356 12815247

[24] McKenna JT Yang C Franciosi S Winston S Abarr KK Rigby MS Distribution and intrinsic membrane properties of basal forebrain GABAergic and parvalbumin neurons in the mouse. J Comp Neurol (2013) 521:1225–50. 10.1002/cne.23290 23254904 PMC3627393

[25] Tamamaki N Yanagawa Y Tomioka R Miyazaki J Obata K Kaneko T Green fluorescent protein expression and colocalization with calretinin, parvalbumin, and somatostatin in the GAD67-GFP knock-in mouse. J Comp Neurol (2003) 467(1):60–79. 10.1002/cne.10905 14574680

[26] Chen L McKenna JT Leonard MZ Yanagawa Y McCarley RW Brown RE. GAD67-GFP knock-in mice have normal sleep-wake patterns and sleep homeostasis. Neuroreport (2010) 21:216–20. 10.1097/WNR.0b013e32833655c4 20051926 PMC3201775

[27] McNally JM McCarley RW McKenna JT Yanagawa Y Brown RE. Complex receptor mediation of acute ketamine application on in vitro gamma oscillations in mouse prefrontal cortex: modeling gamma band oscillation abnormalities in schizophrenia. Neuroscience (2011) 199:51–63. 10.1016/j.neuroscience.2011.10.015 22027237 PMC3237956

[28] Brown RE McKenna JT Winston S Basheer R Yanagawa Y Thakkar MM Characterization of GABAergic neurons in rapid-eye-movement sleep controlling regions of the brainstem reticular formation in GAD67-green fluorescent protein knock-in mice. Eur J Neurosci (2008) 27:352–63. 10.1111/j.1460-9568.2008.06024.x 18215233 PMC2376819

[29] McKenna JT Rigby MS Chen L Winston S Yanagawa Y McCarley RW GAD67-GFP knock-in mice as a tool to investigate GABAergic neurons involved in behavioral state control. Sleep (2010) 33:A136.

[30] Yang C Franciosi S Brown RE. Adenosine inhibits the excitatory synaptic inputs to basal forebrain cholinergic, GABAergic, and parvalbumin neurons in mice. Front Neurol (2013) 4:77. 10.3389/fneur.2013.00077 23801984 PMC3687201

[31] Yang C McKenna JT Zant JC Winston S Basheer R Brown RE. Cholinergic neurons excite cortically projecting basal forebrain GABAergic neurons. J Neurosci (2014) 34:2832–44. 10.1523/JNEUROSCI.3235-13.2014 24553925 PMC3931499

[32] Brown RE Franciosi S McKenna JT Winston S Yanagawa Y McCarley RW Electrophysiological and pharmacological characterization of cortically projecting basal forebrain neurons in the mouse. Soc Neurosci Abs (2008). abstr. 384.16.

[33] Deisseroth K Optogenetics. Nat Methods (2011) 8:26–9. 10.1038/nmeth.f.324 21191368 PMC6814250

[34] Lee HM Giguere PM Roth BL. DREADDs: novel tools for drug discovery and development. Drug Discov Today (2014) 19:469–73. 10.1016/j.drudis.2013.10.018 24184433 PMC4004703

[35] Kim T McKenna JT McNally JM Winston S Yang C Chen L Optogenetic stimulation of parvalbumin-positive basal forebrain neurons entrains cortical gamma oscillations and promotes wakefulness. Soc Neurosci Abs (2011). abstr. 286.215.

[36] Kim T Thankachan S McKenna JT McNally JM Yang C Choi JH Cortically projecting basal forebrain parvalbumin neurons regulate cortical gamma band oscillations. Proc Natl Acad Sci U S A (2015) 112(11):3535–40. 10.1073/pnas.1413625112 25733878 PMC4371918

[37] Anaclet C Fuller PM In vivo interrogation of basal forebrain circuitry regulating arousal. Sleep (2013) 36:A24.

[38] Szymusiak R Alam N Steininger TL McGinty D. Sleep-waking discharge patterns of ventrolateral preoptic/anterior hypothalamic neurons in rats. Brain Res (1998) 803:178–88. 10.1016/S0006-8993(98)00631-3 9729371

[39] Szymusiak R Steininger T Alam N McGinty D. Preoptic area sleep-regulating mechanisms. Arch Ital Biol (2001) 139:77–92. 11256189

[40] Hassani OK Lee MG Jones BE. Melanin-concentrating hormone neurons discharge in a reciprocal manner to orexin neurons across the sleep-wake cycle. Proc Natl Acad Sci U S A (2009) 106:2418–22. 10.1073/pnas.0811400106 19188611 PMC2650171

[41] Xi MC Morales FR Chase MH. A GABAergic pontine reticular system is involved in the control of wakefulness and sleep. Sleep Res Online (1999) 2:43–8. 11382881

[42] Krenzer M Anaclet C Vetrivelan R Wang N Vong L Lowell BB Brainstem and spinal cord circuitry regulating REM sleep and muscle atonia. PLoS One (2011) 6:e24998. 10.1371/journal.pone.0024998 22043278 PMC3197189

[43] Freund TF Buzsaki G Interneurons of the hippocampus. Hippocampus (1996) 6:347–470. 10.1002/(SICI)1098-1063(1996)6:4<347::AID-HIPO1>3.0.CO;2-I 8915675

[44] Cardin JA Carlen M Meletis K Knoblich U Zhang F Deisseroth K Driving fast-spiking cells induces gamma rhythm and controls sensory responses. Nature (2009) 459:663–7. 10.1038/nature08002 19396156 PMC3655711

[45] Sohal VS Zhang F Yizhar O Deisseroth K. Parvalbumin neurons and gamma rhythms enhance cortical circuit performance. Nature (2009) 459:698–702. 10.1038/nature07991 19396159 PMC3969859

[46] Freund TF Antal M. GABA-containing neurons in the septum control inhibitory interneurons in the hippocampus. Nature (1988) 336:170–3. 10.1038/336170a0 3185735

[47] Freund TF Meskenaite V. Gamma-Aminobutyric acid-containing basal forebrain neurons innervate inhibitory interneurons in the neocortex. Proc Natl Acad Sci U S A (1992) 89:738–42. 10.1073/pnas.89.2.738 1731348 PMC48314

[48] Henny P Jones BE. Projections from basal forebrain to prefrontal cortex comprise cholinergic, GABAergic and glutamatergic inputs to pyramidal cells or interneurons. Eur J Neurosci (2008) 27:654–70. 10.1111/j.1460-9568.2008.06029.x 18279318 PMC2426826

[49] Ford B Holmes CJ Mainville L Jones BE. GABAergic neurons in the rat pontomesencephalic tegmentum: codistribution with cholinergic and other tegmental neurons projecting to the posterior lateral hypothalamus. J Comp Neurol (1995) 363:177–96. 8642069 10.1002/cne.903630203

[50] Petsche H Stumpf C Gogolak G [The significance of the rabbit’s septum as a relay station between the midbrain and the hippocampus. I. The control of hippocampus arousal activity by the septum cells.]. Electroencephalogr Clin Neurophysiol (1962) 14:202–11. 10.1016/0013-4694(62)90030-5 14038334

[51] Vertes RP Kocsis B. Brainstem-diencephalo-septohippocampal systems controlling the theta rhythm of the hippocampus. Neuroscience (1997) 81:893–926. 9330355 10.1016/s0306-4522(97)00239-x

[52] Goto M Swanson LW Canteras NS Connections of the nucleus incertus. J Comp Neurol (2001) 438:86–122. 10.1002/cne.1303 11503154

[53] Olucha-Bordonau FE Teruel V Barcia-Gonzalez J Ruiz-Torner A Valverde-Navarro AA Martinez-Soriano F. Cytoarchitecture and efferent projections of the nucleus incertus of the rat. J Comp Neurol (2003) 464:62–97. 10.1002/cne.10774 12866129

[54] Ma S Bonaventure P Ferraro T Shen PJ Burazin TC Bathgate RA Relaxin-3 in GABA projection neurons of nucleus incertus suggests widespread influence on forebrain circuits via G-protein-coupled receptor-135 in the rat. Neuroscience (2007) 144:165–90. 10.1016/j.neuroscience.2006.08.072 17071007

[55] Teruel-Marti V Cervera-Ferri A Nunez A Valverde-Navarro AA Olucha-Bordonau FE Ruiz-Torner A. Anatomical evidence for a ponto-septal pathway via the nucleus incertus in the rat. Brain Res (2008) 1218:87–96. 10.1016/j.brainres.2008.04.022 18514169

[56] Olucha-Bordonau FE Otero-Garcia M Sanchez-Perez AM Nunez A Ma S Gundlach AL. Distribution and targets of the relaxin-3 innervation of the septal area in the rat. J Comp Neurol (2012) 520:1903–39. 10.1002/cne.23018 22134882

[57] Sanchez-Perez AM Arnal-Vicente I Santos FN Pereira CW Elmlili N Sanjuan J Septal projections to nucleus incertus in the rat: bidirectional pathways for modulation of hippocampal function. J Comp Neurol (2015) 523(4):565–88. 10.1002/cne.23687 25269409

[58] Nunez A Cervera-Ferri A Olucha-Bordonau F Ruiz-Torner A Teruel V. Nucleus incertus contribution to hippocampal theta rhythm generation. Eur J Neurosci (2006) 23:2731–8. 10.1111/j.1460-9568.2006.04797.x 16817876

[59] Ma S Blasiak A Olucha-Bordonau FE Verberne AJ Gundlach AL. Heterogeneous responses of nucleus incertus neurons to corticotrophin-releasing factor and coherent activity with hippocampal theta rhythm in the rat. J Physiol (2013) 591:3981–4001. 10.1113/jphysiol.2013.254300 23671163 PMC3764641

[60] Nategh M Nikseresht S Khodagholi F Motamedi F. Nucleus incertus inactivation impairs spatial learning and memory in rats. Physiol Behav (2015) 139:112–20. 10.1016/j.physbeh.2014.11.014 25446222

[61] Von Gudden B Uber das corpus mammillare und die sogenannten schenkel des fornix. Vers Deutsch Natforsch (1884) 57:126.

[62] Hayakawa T Zyo K. Comparative cytoarchitectonic study of Gudden’s tegmental nuclei in some mammals. J Comp Neurol (1983) 216:233–44. 10.1002/cne.902160302 6345599

[63] Wirtshafter D Stratford TR. Evidence for GABAergic projections from the tegmental nuclei of Gudden to the mammillary body in the rat. Brain Res (1993) 630:188–94. 10.1016/0006-8993(93)90656-8 8118685

[64] Saunders RC Vann SD Aggleton JP. Projections from Gudden’s tegmental nuclei to the mammillary body region in the cynomolgus monkey (*Macaca fascicularis*). J Comp Neurol (2012) 520:1128–45. 10.1002/cne.22740 21830220 PMC3909929

[65] Kocsis B Di Prisco GV Vertes RP. Theta synchronization in the limbic system: the role of Gudden’s tegmental nuclei. Eur J Neurosci (2001) 13(2):381–8. 10.1111/j.1460-9568.2001.tb01708.x 11168543

[66] Bassant MH Poindessous-Jazat F. Ventral tegmental nucleus of Gudden: a pontine hippocampal theta generator? Hippocampus (2001) 11:809–13. 10.1002/hipo.1096 11811675

[67] Bassant MH Poindessous-Jazat F. Sleep-related increase in activity of mesopontine neurons in old rats. Neurobiol Aging (2002) 23(4):615–24. 10.1016/S0197-4580(01)00339-6 12009510

[68] Brown RE Franciosi S Yanagawa Y McCarley RW Cellular mechanisms underlying theta rhythm in a mammillary body-tegmentum circuit. Soc Neurosci Abs (2007). abstr. 734.715.

[69] Brown RE McKenna JT Winston S Yanagawa Y McCarley RW Long-lasting plateau potentials and carbachol suppression of orexin excitation in GABAergic ventral tegmental nucleus of Gudden neurons: implications for theta burst firing. Sleep (2009) 32:A32.

[70] Hayakawa T Zyo K. Retrograde double-labeling study of the mammillothalamic and the mammillotegmental projections in the rat. J Comp Neurol (1989) 284:1–11. 10.1002/cne.902840102 2502564

[71] Allen GV Hopkins DA. Topography and synaptology of mamillary body projections to the mesencephalon and pons in the rat. J Comp Neurol (1990) 301:214–31. 10.1002/cne.903010206 1702105

[72] McKenna JT Franciosi S Winston S Yanagawa Y McCarley RW Brown RE Neuroanatomical investigation of brainstem projections to the medial mammillary body:possible implications for the modulation of theta rhythm. Sleep (2009) 32:A30.

[73] Vann SD Aggleton JP The mammillary bodies: two memory systems in one? Nat Rev Neurosci (2004) 5:35–44. 10.1038/nrn1299 14708002

[74] Kirk IJ Oddie SD Konopacki J Bland BH. Evidence for differential control of posterior hypothalamic, supramammillary, and medial mammillary theta-related cellular discharge by ascending and descending pathways. J Neurosci (1996) 16:5547–54. 8757266 10.1523/JNEUROSCI.16-17-05547.1996 PMC6578895

[75] Kocsis B Vertes RP. Phase relations of rhythmic neuronal firing in the supramammillary nucleus and mammillary body to the hippocampal theta activity in urethane anesthetized rats. Hippocampus (1997) 7:204–14. 10.1002/(SICI)1098-1063(1997)7:2<204::AID-HIPO7>3.0.CO;2-M 9136050

[76] Alonso A Llinas RR. Electrophysiology of the mammillary complex in vitro. II. Medial mammillary neurons. J Neurophysiol (1992) 68:1321–31. 1432086 10.1152/jn.1992.68.4.1321

[77] Swanson LW Cowan WM. Hippocampo-hypothalamic connections: origin in subicular cortex, not ammon’s horn. Science (1975) 189:303–4. 10.1126/science.49928 49928

[78] Kirk IJ Mackay JC. The role of theta-range oscillations in synchronising and integrating activity in distributed mnemonic networks. Cortex (2003) 39:993–1008. 10.1016/S0010-9452(08)70874-8 14584563

[79] Vertes RP Albo Z Viana DP. Theta-rhythmically firing neurons in the anterior thalamus: implications for mnemonic functions of Papez’s circuit. Neuroscience (2001) 104:619–25. 10.1016/S0306-4522(01)00131-2 11440795

[80] Tsanov M Chah E Wright N Vann SD Reilly R Erichsen JT Oscillatory entrainment of thalamic neurons by theta rhythm in freely moving rats. J Neurophysiol (2011) 105:4–17. 10.1152/jn.00771.2010 20962067 PMC3023377

[81] Papez JW A proposed mechanism of emotion. 1937. J Neuropsychiatry Clin Neurosci (1995) 7:103–12. 10.1176/jnp.7.1.103 7711480

[82] Vann SD. Dismantling the Papez circuit for memory in rats. Elife (2013) 2:e00736. 10.7554/eLife.00736 23805381 PMC3691571

[83] Vann SD. Re-evaluating the role of the mammillary bodies in memory. Neuropsychologia (2010) 48:2316–27. 10.1016/j.neuropsychologia.2009.10.019 19879886

[84] Vann SD. Gudden’s ventral tegmental nucleus is vital for memory: re-evaluating diencephalic inputs for amnesia. Brain (2009) 132:2372–84. 10.1093/brain/awp175 19602577

[85] Tsanov M Chah E Vann SD Reilly RB Erichsen JT Aggleton JP Theta-modulated head direction cells in the rat anterior thalamus. J Neurosci (2011) 31:9489–502. 10.1523/JNEUROSCI.0353-11.2011 21715614 PMC3855197

[86] Tsanov M O’Mara SM. Decoding signal processing in thalamo-hippocampal circuitry: implications for theories of memory and spatial processing. Brain Res (2014). 10.1016/j.brainres.2014.12.003 25498107

[87] Carr DB Sesack SR. GABA-containing neurons in the rat ventral tegmental area project to the prefrontal cortex. Synapse (2000) 38:114–23. 10.1002/1098-2396(200011)38:2<114::AID-SYN2>3.0.CO;2-R 11018785

[88] Nair-Roberts RG Chatelain-Badie SD Benson E White-Cooper H Bolam JP Ungless MA. Stereological estimates of dopaminergic, GABAergic and glutamatergic neurons in the ventral tegmental area, substantia nigra and retrorubral field in the rat. Neuroscience (2008) 152:1024–31. 10.1016/j.neuroscience.2008.01.046 18355970 PMC2575227

[89] Chieng B Azriel Y Mohammadi S Christie MJ. Distinct cellular properties of identified dopaminergic and GABAergic neurons in the mouse ventral tegmental area. J Physiol (2011) 589:3775–87. 10.1113/jphysiol.2011.210807 21646409 PMC3171885

[90] Taylor SR Badurek S Dileone RJ Nashmi R Minichiello L Picciotto MR. GABAergic and glutamatergic efferents of the mouse ventral tegmental area. J Comp Neurol (2014) 522:3308–34. 10.1002/cne.23603 24715505 PMC4107038

[91] Pirot S Godbout R Mantz J Tassin JP Glowinski J Thierry AM. Inhibitory effects of ventral tegmental area stimulation on the activity of prefrontal cortical neurons: evidence for the involvement of both dopaminergic and GABAergic components. Neuroscience (1992) 49:857–65. 10.1016/0306-4522(92)90362-6 1436485

[92] Van Bockstaele EJ Pickel VM. GABA-containing neurons in the ventral tegmental area project to the nucleus accumbens in rat brain. Brain Res (1995) 682:215–21. 10.1016/0006-8993(95)00334-M 7552315

[93] Steffensen SC Svingos AL Pickel VM Henriksen SJ. Electrophysiological characterization of GABAergic neurons in the ventral tegmental area. J Neurosci (1998) 18:8003–15. 9742167 10.1523/JNEUROSCI.18-19-08003.1998 PMC6793009

[94] Brown MT Tan KR O’connor EC Nikonenko I Muller D Luscher C. Ventral tegmental area GABA projections pause accumbal cholinergic interneurons to enhance associative learning. Nature (2012) 492:452–6. 10.1038/nature11657 23178810

[95] Stobbs SH Ohran AJ Lassen MB Allison DW Brown JE Steffensen SC. Ethanol suppression of ventral tegmental area GABA neuron electrical transmission involves N-methyl-D-aspartate receptors. J Pharmacol Exp Ther (2004) 311:282–9. 10.1124/jpet.104.071860 15169831

[96] Allison DW Ohran AJ Stobbs SH Mameli M Valenzuela CF Sudweeks SN Connexin-36 gap junctions mediate electrical coupling between ventral tegmental area GABA neurons. Synapse (2006) 60:20–31. 10.1002/syn.20272 16575850

[97] Lassen MB Brown JE Stobbs SH Gunderson SH Maes L Valenzuela CF Brain stimulation reward is integrated by a network of electrically coupled GABA neurons. Brain Res (2007) 1156:46–58. 10.1016/j.brainres.2007.04.053 17524371 PMC4056590

[98] Lee RS Steffensen SC Henriksen SJ. Discharge profiles of ventral tegmental area GABA neurons during movement, anesthesia, and the sleep-wake cycle. J Neurosci (2001) 21(5):1757–66. 11222665 10.1523/JNEUROSCI.21-05-01757.2001 PMC6762953

[99] Steffensen SC Lee RS Stobbs SH Henriksen SJ. Responses of ventral tegmental area GABA neurons to brain stimulation reward. Brain Res (2001) 906:190–7. 10.1016/S0006-8993(01)02581-1 11430879

[100] Korotkova TM Sergeeva OA Eriksson KS Haas HL Brown RE. Excitation of ventral tegmental area dopaminergic and nondopaminergic neurons by orexins/hypocretins. J Neurosci (2003) 23(1):7–11. 12514194 10.1523/JNEUROSCI.23-01-00007.2003 PMC6742159

[101] Korotkova TM Brown RE Sergeeva OA Ponomarenko AA Haas HL. Effects of arousal- and feeding-related neuropeptides on dopaminergic and GABAergic neurons in the ventral tegmental area of the rat. Eur J Neurosci (2006) 23:2677–85. 10.1111/j.1460-9568.2006.04977.x 16817870

[102] Korotkova TM Haas HL Brown RE. Histamine excites GABAergic cells in the rat substantia nigra and ventral tegmental area in vitro. Neurosci Lett (2002) 320:133–6. 10.1016/S0304-3940(02)00050-2 11852180

[103] Carr DB Sesack SR. Projections from the rat prefrontal cortex to the ventral tegmental area: target specificity in the synaptic associations with mesoaccumbens and mesocortical neurons. J Neurosci (2000) 20(10):3864–73. 10804226 10.1523/JNEUROSCI.20-10-03864.2000 PMC6772693

[104] Fujisawa S Buzsaki G. A 4 Hz oscillation adaptively synchronizes prefrontal, VTA, and hippocampal activities. Neuron (2011) 72:153–65. 10.1016/j.neuron.2011.08.018 21982376 PMC3235795

[105] Johnson SW North RA. Opioids excite dopamine neurons by hyperpolarization of local interneurons. J Neurosci (1992) 12:483–8. 1346804 10.1523/JNEUROSCI.12-02-00483.1992 PMC6575608

[106] Tan KR Brown M Labouebe G Yvon C Creton C Fritschy JM Neural bases for addictive properties of benzodiazepines. Nature (2010) 463:769–74. 10.1038/nature08758 20148031 PMC2871668

[107] Van Zessen R Phillips JL Budygin EA Stuber GD. Activation of VTA GABA neurons disrupts reward consumption. Neuron (2012) 73:1184–94. 10.1016/j.neuron.2012.02.016 22445345 PMC3314244

[108] Cohen JY Haesler S Vong L Lowell BB Uchida N. Neuron-type-specific signals for reward and punishment in the ventral tegmental area. Nature (2012) 482:85–8. 10.1038/nature10754 22258508 PMC3271183

[109] Jhou TC Geisler S Marinelli M Degarmo BA Zahm DS. The mesopontine rostromedial tegmental nucleus: a structure targeted by the lateral habenula that projects to the ventral tegmental area of Tsai and substantia nigra compacta. J Comp Neurol (2009) 513:566–96. 10.1002/cne.21891 19235216 PMC3116663

[110] Kaufling J Veinante P Pawlowski SA Freund-Mercier MJ Barrot M. Afferents to the GABAergic tail of the ventral tegmental area in the rat. J Comp Neurol (2009) 513:597–621. 10.1002/cne.21983 19235223

[111] Hong S Jhou TC Smith M Saleem KS Hikosaka O. Negative reward signals from the lateral habenula to dopamine neurons are mediated by rostromedial tegmental nucleus in primates. J Neurosci (2011) 31:11457–71. 10.1523/JNEUROSCI.1384-11.2011 21832176 PMC3315151

[112] Barrot M Sesack SR Georges F Pistis M Hong S Jhou TC. Braking dopamine systems: a new GABA master structure for mesolimbic and nigrostriatal functions. J Neurosci (2012) 32:14094–101. 10.1523/JNEUROSCI.3370-12.2012 23055478 PMC3513755

[113] Matsumoto M Hikosaka O. Lateral habenula as a source of negative reward signals in dopamine neurons. Nature (2007) 447:1111–5. 10.1038/nature05860 17522629

[114] Jhou TC Fields HL Baxter MG Saper CB Holland PC. The rostromedial tegmental nucleus (RMTg), a GABAergic afferent to midbrain dopamine neurons, encodes aversive stimuli and inhibits motor responses. Neuron (2009) 61:786–800. 10.1016/j.neuron.2009.02.001 19285474 PMC2841475

[115] Lecca S Melis M Luchicchi A Muntoni AL Pistis M. Inhibitory inputs from rostromedial tegmental neurons regulate spontaneous activity of midbrain dopamine cells and their responses to drugs of abuse. Neuropsychopharmacology (2012) 37:1164–76. 10.1038/npp.2011.302 22169942 PMC3306878

[116] Jalabert M Bourdy R Courtin J Veinante P Manzoni OJ Barrot M Neuronal circuits underlying acute morphine action on dopamine neurons. Proc Natl Acad Sci U S A (2011) 108:16446–50. 10.1073/pnas.1105418108 21930931 PMC3182694

[117] Saper CB. Organization of cerebral cortical afferent systems in the rat. II. Hypothalamocortical projections. J Comp Neurol (1985) 237:21–46. 10.1002/cne.902370103 2995455

[118] Brown RE Stevens DR Haas HL The physiology of brain histamine. Prog Neurobiol (2001) 63(6):637–72. 10.1016/S0301-0082(00)00039-3 11164999

[119] Esclapez M Tillakaratne NJ Tobin AJ Houser CR. Comparative localization of mRNAs encoding two forms of glutamic acid decarboxylase with nonradioactive in situ hybridization methods. J Comp Neurol (1993) 331:339–62. 10.1002/cne.903310305 8514913

[120] Williams RH Chee MJ Kroeger D Ferrari LL Maratos-Flier E Scammell TE Optogenetic-mediated release of histamine reveals distal and autoregulatory mechanisms for controlling arousal. J Neurosci (2014) 34:6023–9. 10.1523/JNEUROSCI.4838-13.2014 24760861 PMC3996219

[121] Lin CS Nicolelis MA Schneider JS Chapin JK. A major direct GABAergic pathway from zona incerta to neocortex. Science (1990) 248:1553–6. 10.1126/science.2360049 2360049

[122] Kohler C Haglund L Swanson LW. A diffuse alpha MSH-immunoreactive projection to the hippocampus and spinal cord from individual neurons in the lateral hypothalamic area and zona incerta. J Comp Neurol (1984) 223:501–14. 10.1002/cne.902230404 6325509

[123] Shiosaka S Shibasaki T Tohyama M. Bilateral alpha-melanocyte stimulating hormonergic fiber system from zona incerta to cerebral cortex: combined retrograde axonal transport and immunohistochemical study. Brain Res (1984) 309:350–3. 6383519 10.1016/0006-8993(84)90602-4

[124] Saper CB Akil H Watson SJ. Lateral hypothalamic innervation of the cerebral cortex: immunoreactive staining for a peptide resembling but immunochemically distinct from pituitary/arcuate alpha-melanocyte stimulating hormone. Brain Res Bull (1986) 16:107–20. 2420417 10.1016/0361-9230(86)90018-3

[125] Bartho P Freund TF Acsady L. Selective GABAergic innervation of thalamic nuclei from zona incerta. Eur J Neurosci (2002) 16:999–1014. 10.1046/j.1460-9568.2002.02157.x 12383229

[126] Trageser JC Burke KA Masri R Li Y Sellers L Keller A. State-dependent gating of sensory inputs by zona incerta. J Neurophysiol (2006) 96:1456–63. 10.1152/jn.00423.2006 16775205 PMC1764852

[127] Lavallee P Urbain N Dufresne C Bokor H Acsady L Deschenes M. Feedforward inhibitory control of sensory information in higher-order thalamic nuclei. J Neurosci (2005) 25:7489–98. 10.1523/JNEUROSCI.2301-05.2005 16107636 PMC2670454

[128] Sidibe M Bevan MD Bolam JP Smith Y. Efferent connections of the internal globus pallidus in the squirrel monkey: I. Topography and synaptic organization of the pallidothalamic projection. J Comp Neurol (1997) 382:323–47. 10.1002/(SICI)1096-9861(19970609)382:3<323::AID-CNE3>3.0.CO;2-5 9183697

[129] Kha HT Finkelstein DI Tomas D Drago J Pow DV Horne MK. Projections from the substantia nigra pars reticulata to the motor thalamus of the rat: single axon reconstructions and immunohistochemical study. J Comp Neurol (2001) 440:20–30. 10.1002/cne.1367 11745605

[130] Sidibe M Pare JF Smith Y. Nigral and pallidal inputs to functionally segregated thalamostriatal neurons in the centromedian/parafascicular intralaminar nuclear complex in monkey. J Comp Neurol (2002) 447(3):286–99. 10.1002/cne.10247 11984822

[131] Churchill L Zahm DS Kalivas PW. The mediodorsal nucleus of the thalamus in rats – I. forebrain gabaergic innervation. Neuroscience (1996) 70:93–102. 10.1016/0306-4522(95)00351-I 8848140

[132] Steriade M Deschenes M Domich L Mulle C. Abolition of spindle oscillations in thalamic neurons disconnected from nucleus reticularis thalami. J Neurophysiol (1985) 54:1473–97. 4087044 10.1152/jn.1985.54.6.1473

[133] Halassa MM Siegle JH Ritt JT Ting JT Feng G Moore CI. Selective optical drive of thalamic reticular nucleus generates thalamic bursts and cortical spindles. Nat Neurosci (2011) 14:1118–20. 10.1038/nn.2880 21785436 PMC4169194

[134] Mukhametov LM Rizzolatti G Tradardi V. Spontaneous activity of neurones of nucleus reticularis thalami in freely moving cats. J Physiol (1970) 210:651–67. 10.1113/jphysiol.1970.sp009233 5499817 PMC1395618

[135] Barrionuevo G Benoit O Tempier P Evidence for two types of firing pattern during the sleep-waking cycle in the reticular thalamic nucleus of the cat. Exp Neurol (1981) 72:486–501. 10.1016/0014-4886(81)90238-7 7238704

[136] Steriade M Domich L Oakson G. Reticularis thalami neurons revisited: activity changes during shifts in states of vigilance. J Neurosci (1986) 6:68–81. 3944624 10.1523/JNEUROSCI.06-01-00068.1986 PMC6568617

[137] McCormick DA Wang Z. Serotonin and noradrenaline excite GABAergic neurones of the guinea-pig and cat nucleus reticularis thalami. J Physiol (1991) 442:235–55. 10.1113/jphysiol.1991.sp018791 1665858 PMC1179887

[138] Halassa MM Chen Z Wimmer RD Brunetti PM Zhao S Zikopoulos B State-dependent architecture of thalamic reticular subnetworks. Cell (2014) 158:808–21. 10.1016/j.cell.2014.06.025 25126786 PMC4205482

[139] McAlonan K Cavanaugh J Wurtz RH. Guarding the gateway to cortex with attention in visual thalamus. Nature (2008) 456:391–4. 10.1038/nature07382 18849967 PMC2713033

[140] Kayahara T Nakano K. The globus pallidus sends axons to the thalamic reticular nucleus neurons projecting to the centromedian nucleus of the thalamus: a light and electron microscope study in the cat. Brain Res Bull (1998) 45:623–30. 10.1016/S0361-9230(97)00464-4 9566507

[141] Gulcebi MI Ketenci S Linke R Hacioglu H Yanali H Veliskova J Topographical connections of the substantia nigra pars reticulata to higher-order thalamic nuclei in the rat. Brain Res Bull (2012) 87:312–8. 10.1016/j.brainresbull.2011.11.005 22108631

[142] Jourdain A Semba K Fibiger HC. Basal forebrain and mesopontine tegmental projections to the reticular thalamic nucleus: an axonal collateralization and immunohistochemical study in the rat. Brain Res (1989) 505:55–65. 10.1016/0006-8993(89)90115-7 2575437

[143] Bickford ME Gunluk AE Van Horn SC Sherman SM. GABAergic projection from the basal forebrain to the visual sector of the thalamic reticular nucleus in the cat. J Comp Neurol (1994) 348:481–510. 7836559 10.1002/cne.903480402

[144] Zaborszky L Gaykema RP Swanson DJ Cullinan WE Cortical input to the basal forebrain. Neuroscience (1997) 79:1051–78. 10.1016/S0306-4522(97)00049-3 9219967

[145] Golmayo L Nunez A Zaborszky L. Electrophysiological evidence for the existence of a posterior cortical-prefrontal-basal forebrain circuitry in modulating sensory responses in visual and somatosensory rat cortical areas. Neuroscience (2003) 119:597–609. 10.1016/S0306-4522(03)00031-9 12770572

[146] Gyengesi E Zaborszky L Detari L. The effect of prefrontal stimulation on the firing of basal forebrain neurons in urethane anesthetized rat. Brain Res Bull (2008) 75:570–80. 10.1016/j.brainresbull.2007.09.008 18355633 PMC2423328

[147] Pinault D Deschenes M. Control of 40-Hz firing of reticular thalamic cells by neurotransmitters. Neuroscience (1992) 51:259–68. 10.1016/0306-4522(92)90313-Q 1361219

[148] Pinault D Deschenes M. Voltage-dependent 40-Hz oscillations in rat reticular thalamic neurons in vivo. Neuroscience (1992) 51:245–58. 10.1016/0306-4522(92)90312-P 1465191

[149] MacDonald KD Fifkova E Jones MS Barth DS. Focal stimulation of the thalamic reticular nucleus induces focal gamma waves in cortex. J Neurophysiol (1998) 79:474–7. 9425216 10.1152/jn.1998.79.1.474

[150] Van Der Werf YD Witter MP Groenewegen HJ. The intralaminar and midline nuclei of the thalamus. Anatomical and functional evidence for participation in processes of arousal and awareness. Brain Res Brain Res Rev (2002) 39:107–40. 10.1016/S0165-0173(02)00181-9 12423763

[151] Alkire MT McReynolds JR Hahn EL Trivedi AN. Thalamic microinjection of nicotine reverses sevoflurane-induced loss of righting reflex in the rat. Anesthesiology (2007) 107:264–72. 10.1097/01.anes.0000270741.33766.24 17667571

[152] Alkire MT Asher CD Franciscus AM Hahn EL. Thalamic microinfusion of antibody to a voltage-gated potassium channel restores consciousness during anesthesia. Anesthesiology (2009) 110:766–73. 10.1097/ALN.0b013e31819c461c 19322942

[153] Quinkert AW Schiff ND Pfaff DW. Temporal patterning of pulses during deep brain stimulation affects central nervous system arousal. Behav Brain Res (2010) 214:377–85. 10.1016/j.bbr.2010.06.009 20558210

[154] Lioudyno MI Birch AM Tanaka BS Sokolov Y Goldin AL Chandy KG Shaker-related potassium channels in the central medial nucleus of the thalamus are important molecular targets for arousal suppression by volatile general anesthetics. J Neurosci (2013) 33:16310–22. 10.1523/JNEUROSCI.0344-13.2013 24107962 PMC3792466

[155] Baker R Gent TC Yang Q Parker S Vyssotski AL Wisden W Altered activity in the central medial thalamus precedes changes in the neocortex during transitions into both sleep and propofol anesthesia. J Neurosci (2014) 34:13326–35. 10.1523/JNEUROSCI.1519-14.2014 25274812 PMC4180471

[156] Duncan GE Breese GR Criswell HE McCown TJ Herbert JS Devaud LL Distribution of [3H]zolpidem binding sites in relation to messenger RNA encoding the alpha 1, beta 2 and gamma 2 subunits of GABAA receptors in rat brain. Neuroscience (1995) 64:1113–28. 10.1016/0306-4522(94)00433-6 7753379

[157] Fritschy JM Mohler H. GABAA-receptor heterogeneity in the adult rat brain: differential regional and cellular distribution of seven major subunits. J Comp Neurol (1995) 359:154–94. 10.1002/cne.903590111 8557845

[158] Chen L Savio CC Yung WH. Electrophysiological and behavioral effects of zolpidem in rat globus pallidus. Exp Neurol (2004) 186:212–20. 10.1016/j.expneurol.2003.11.003 15026257

[159] Zhang LL Chen L Xue Y Yung WH. Modulation of synaptic GABAA receptor function by zolpidem in substantia nigra pars reticulata. Acta Pharmacol Sin (2008) 29:161–8. 10.1111/j.1745-7254.2008.00735.x 18215344

[160] Dennis T Dubois A Benavides J Scatton B. Distribution of central omega 1 (benzodiazapine1) and omega 2 (benzodiazapine2) receptor subtypes in the monkey and human brain. An autoradiographic study with [3H]flunitrazepam and the omega 1 selective ligand [3H]zolpidem. J Pharmacol Exp Ther (1988) 247(1):309–22. 2845057

[161] Schiff ND. Recovery of consciousness after brain injury: a mesocircuit hypothesis. Trends Neurosci (2010) 33:1–9. 10.1016/j.tins.2009.11.002 19954851 PMC2931585

[162] Brefel-Courbon C Payoux P Ory F Sommet A Slaoui T Raboyeau G Clinical and imaging evidence of zolpidem effect in hypoxic encephalopathy. Ann Neurol (2007) 62(1):102–5. 10.1002/ana.21110 17357126

[163] Whyte J Myers R. Incidence of clinically significant responses to zolpidem among patients with disorders of consciousness: a preliminary placebo controlled trial. Am J Phys Med Rehabil (2009) 88:410–8. 10.1097/PHM.0b013e3181a0e3a0 19620954

[164] Hall SD Yamawaki N Fisher AE Clauss RP Woodhall GL Stanford IM. GABA(A) alpha-1 subunit mediated desynchronization of elevated low frequency oscillations alleviates specific dysfunction in stroke – a case report. Clin Neurophysiol (2010) 121:549–55. 10.1016/j.clinph.2009.11.084 20097125

[165] Machado C Estevez M Perez-Nellar J Gutierrez J Rodriguez R Carballo M Autonomic, EEG, and behavioral arousal signs in a PVS case after Zolpidem intake. Can J Neurol Sci (2011) 38:341–4. 10.1017/S0317167100011562 21320843

[166] Whyte J Rajan R Rosenbaum A Katz D Kalmar K Seel R Zolpidem and restoration of consciousness. Am J Phys Med Rehabil (2014) 93:101–13. 10.1097/PHM.0000000000000069 24434886

[167] Nicolelis MA Chapin JK Lin RC. Somatotopic maps within the zona incerta relay parallel GABAergic somatosensory pathways to the neocortex, superior colliculus, and brainstem. Brain Res (1992) 577:134–41. 10.1016/0006-8993(92)90546-L 1521138

[168] Urbain N Deschenes M. Motor cortex gates vibrissal responses in a thalamocortical projection pathway. Neuron (2007) 56:714–25. 10.1016/j.neuron.2007.10.023 18031687

[169] Bartho P Slezia A Varga V Bokor H Pinault D Buzsaki G Cortical control of zona incerta. J Neurosci (2007) 27:1670–81. 10.1523/JNEUROSCI.3768-06.2007 17301175 PMC2670453

[170] Gritti I Mariotti M Mancia M. GABAergic and cholinergic basal forebrain and preoptic-anterior hypothalamic projections to the mediodorsal nucleus of the thalamus in the cat. Neuroscience (1998) 85:149–78. 10.1016/S0306-4522(97)00573-3 9607710

[171] Metherate R Cox CL Ashe JH. Cellular bases of neocortical activation: modulation of neural oscillations by the nucleus basalis and endogenous acetylcholine. J Neurosci (1992) 12:4701–11. 1361197 10.1523/JNEUROSCI.12-12-04701.1992 PMC6575759

[172] Grothe M Heinsen H Teipel SJ. Atrophy of the cholinergic basal forebrain over the adult age range and in early stages of Alzheimer’s disease. Biol Psychiatry (2012) 71:805–13. 10.1016/j.biopsych.2011.06.019 21816388 PMC3701122

[173] Freund TF Gulyas AI. GABAergic interneurons containing calbindin D28K or somatostatin are major targets of GABAergic basal forebrain afferents in the rat neocortex. J Comp Neurol (1991) 314:187–99. 1686776 10.1002/cne.903140117

[174] Henderson Z Fiddler G Saha S Boros A Halasy K. A parvalbumin-containing, axosomatic synaptic network in the rat medial septum: relevance to rhythmogenesis. Eur J Neurosci (2004) 19:2753–68. 10.1111/j.0953-816X.2004.03399.x 15147309

[175] Korotkova T Fuchs EC Ponomarenko A Von EJ Monyer H. NMDA receptor ablation on parvalbumin-positive interneurons impairs hippocampal synchrony, spatial representations, and working memory. Neuron (2010) 68:557–69. 10.1016/j.neuron.2010.09.017 21040854

[176] Furuta T Koyano K Tomioka R Yanagawa Y Kaneko T. GABAergic basal forebrain neurons that express receptor for neurokinin B and send axons to the cerebral cortex. J Comp Neurol (2004) 473(1):43–58. 10.1002/cne.20087 15067717

[177] Hermanstyne TO Kihira Y Misono K Deitchler A Yanagawa Y Misonou H. Immunolocalization of the voltage-gated potassium channel Kv2.2 in GABAergic neurons in the basal forebrain of rats and mice. J Comp Neurol (2010) 518:4298–310. 10.1002/cne.22457 20853508 PMC3005293

[178] Hermanstyne TO Subedi K Le WW Hoffman GE Meredith AL Mong JA Kv2.2: a novel molecular target to study the role of basal forebrain GABAergic neurons in the sleep-wake cycle. Sleep (2013) 36:1839–48. 10.5665/sleep.3212 24293758 PMC3825433

[179] Borhegyi Z Varga V Szilagyi N Fabo D Freund TF. Phase segregation of medial septal GABAergic neurons during hippocampal theta activity. J Neurosci (2004) 24(39):8470–9. 10.1523/JNEUROSCI.1413-04.2004 15456820 PMC6729892

[180] Simon AP Poindessous-Jazat F Dutar P Epelbaum J Bassant MH. Firing properties of anatomically identified neurons in the medial septum of anesthetized and unanesthetized restrained rats. J Neurosci (2006) 26:9038–46. 10.1523/JNEUROSCI.1401-06.2006 16943562 PMC6675331

[181] Maloney KJ Cape EG Gotman J Jones BE. High-frequency gamma electroencephalogram activity in association with sleep-wake states and spontaneous behaviors in the rat. Neuroscience (1997) 76:541–55. 10.1016/S0306-4522(96)00298-9 9015337

[182] Gross DW Gotman J. Correlation of high-frequency oscillations with the sleep-wake cycle and cognitive activity in humans. Neuroscience (1999) 94:1005–18. 10.1016/S0306-4522(99)00343-7 10625043

[183] Morris NP Harris SJ Henderson Z. Parvalbumin-immunoreactive, fast-spiking neurons in the medial septum/diagonal band complex of the rat: intracellular recordings in vitro. Neuroscience (1999) 92:589–600. 10.1016/S0306-4522(99)00026-3 10408608

[184] Henderson Z Lu CB Janzso G Matto N McKinley CE Yanagawa Y Distribution and role of Kv3.1b in neurons in the medial septum diagonal band complex. Neuroscience (2010) 166:952–69. 10.1016/j.neuroscience.2010.01.020 20083165

[185] Rudy B Chow A Lau D Amarillo Y Ozaita A Saganich M Contributions of Kv3 channels to neuronal excitability. Ann N Y Acad Sci (1999) 868:304–43. 10.1111/j.1749-6632.1999.tb11295.x 10414303

[186] Kocsis B Li S. In vivo contribution of h-channels in the septal pacemaker to theta rhythm generation. Eur J Neurosci (2004) 20:2149–58. 10.1111/j.1460-9568.2004.03678.x 15450094

[187] Xu C Datta S Wu M Alreja M. Hippocampal theta rhythm is reduced by suppression of the H-current in septohippocampal GABAergic neurons. Eur J Neurosci (2004) 19:2299–309. 10.1111/j.0953-816X.2004.03316.x 15090056

[188] Furuta T Mori T Lee T Kaneko T. Third group of neostriatofugal neurons: neurokinin B-producing neurons that send axons predominantly to the substantia innominata. J Comp Neurol (2000) 426:279–96. 10.1002/1096-9861(20001016)426:2<279::AID-CNE9>3.0.CO;2-F 10982469

[189] Navarro VM Ruiz-Pino F Sanchez-Garrido MA Garcia-Galiano D Hobbs SJ Manfredi-Lozano M Role of neurokinin B in the control of female puberty and its modulation by metabolic status. J Neurosci (2012) 32:2388–97. 10.1523/JNEUROSCI.4288-11.2012 22396413 PMC3567461

[190] Wu M Shanabrough M Leranth C Alreja M. Cholinergic excitation of septohippocampal GABA but not cholinergic neurons: implications for learning and memory. J Neurosci (2000) 20(10):3900–8. 10804229 10.1523/JNEUROSCI.20-10-03900.2000 PMC6772671

[191] Liu W Kumar A Alreja M. Excitatory effects of muscarine on septohippocampal neurons: involvement of M3 receptors. Brain Res (1998) 805:220–33. 9733970 10.1016/s0006-8993(98)00729-x

[192] Wu M Hajszan T Leranth C Alreja M. Nicotine recruits a local glutamatergic circuit to excite septohippocampal GABAergic neurons. Eur J Neurosci (2003) 18:1155–68. 10.1046/j.1460-9568.2003.02847.x 12956714

[193] Zant JC Kim T Kalinchuk AV Yang C Brown RE McNally JM Optogenetic stimulation of basal forebrain cholinergic neurons promotes cortical activation both directly and indirectly. Soc Neurosci Abs (2014). abstr. 257.208.

[194] Alreja M Liu W. Noradrenaline induces IPSCs in rat medial septal/diagonal band neurons: involvement of septohippocampal GABAergic neurons. J Physiol (1996) 494(Pt 1):201–15. 10.1113/jphysiol.1996.sp021485 8814616 PMC1160624

[195] Xu C Michelsen KA Wu M Morozova E Panula P Alreja M. Histamine innervation and activation of septohippocampal GABAergic neurones: involvement of local ACh release. J Physiol (2004) 561:657–70. 10.1113/jphysiol.2004.071712 15486020 PMC1665378

[196] Alreja M. Excitatory actions of serotonin on GABAergic neurons of the medial septum and diagonal band of Broca. Synapse (1996) 22:15–27. 10.1002/(SICI)1098-2396(199601)22:1<15::AID-SYN2>3.0.CO;2-L 8822474

[197] Wu M Zhang Z Leranth C Xu C Van Den Pol AN Alreja M. Hypocretin increases impulse flow in the septohippocampal GABAergic pathway: implications for arousal via a mechanism of hippocampal disinhibition. J Neurosci (2002) 22(17):7754–65. 12196599 10.1523/JNEUROSCI.22-17-07754.2002 PMC6757957

[198] Wu M Zaborszky L Hajszan T Van Den Pol AN Alreja M. Hypocretin/orexin innervation and excitation of identified septohippocampal cholinergic neurons. J Neurosci (2004) 24:3527–36. 10.1523/JNEUROSCI.5364-03.2004 15071100 PMC6729747

[199] Gaykema RP Zaborszky L. Parvalbumin-containing neurons in the basal forebrain receive direct input from the substantia nigra-ventral tegmental area. Brain Res (1997) 747:173–9. 10.1016/S0006-8993(96)01309-1 9042545

[200] Joho RH Ho CS Marks GA. Increased gamma- and decreased delta-oscillations in a mouse deficient for a potassium channel expressed in fast-spiking interneurons. J Neurophysiol (1999) 82:1855–64. 10515974 10.1152/jn.1999.82.4.1855

[201] Espinosa F Marks G Heintz N Joho RH. Increased motor drive and sleep loss in mice lacking Kv3-type potassium channels. Genes Brain Behav (2004) 3:90–100. 10.1046/j.1601-183x.2003.00054.x 15005717

[202] Joho RH Marks GA Espinosa F. Kv3 potassium channels control the duration of different arousal states by distinct stochastic and clock-like mechanisms. Eur J Neurosci (2006) 23:1567–74. 10.1111/j.1460-9568.2006.04672.x 16553620

[203] Espinosa F Torres-Vega MA Marks GA Joho RH. Ablation of Kv3.1 and Kv3.3 potassium channels disrupts thalamocortical oscillations in vitro and in vivo. J Neurosci (2008) 28:5570–81. 10.1523/JNEUROSCI.0747-08.2008 18495891 PMC3844809

[204] Suntsova N Szymusiak R Alam MN Guzman-Marin R McGinty D. Sleep-waking discharge patterns of median preoptic nucleus neurons in rats. J Physiol (2002) 543(2):665–77. 10.1113/jphysiol.2002.023085 12205198 PMC2290500

[205] Takahashi K Lin JS Sakai K. Characterization and mapping of sleep-waking specific neurons in the basal forebrain and preoptic hypothalamus in mice. Neuroscience (2009) 161:269–92. 10.1016/j.neuroscience.2009.02.075 19285545

[206] Sakai K. Sleep-waking discharge profiles of median preoptic and surrounding neurons in mice. Neuroscience (2011) 182:144–61. 10.1016/j.neuroscience.2011.03.010 21396987

[207] Zecharia AY Yu X Gotz T Ye Z Carr DR Wulff P GABAergic inhibition of histaminergic neurons regulates active waking but not the sleep-wake switch or propofol-induced loss of consciousness. J Neurosci (2012) 32:13062–75. 10.1523/JNEUROSCI.2931-12.2012 22993424 PMC3466043

[208] Ciccarelli A Calza A Panzanelli P Concas A Giustetto M Sassoe-Pognetto M. Organization of GABAergic synaptic circuits in the rat ventral tegmental area. PLoS One (2012) 7:e46250. 10.1371/journal.pone.0046250 23056271 PMC3466259

[209] Gao B Hornung J-P Fritschy J-M Identification of distinct GABAA-receptor subtypes in cholinergic and parvalbumin-positive neurons of the rat and marmoset medial septum – diagonal band complex. Neuroscience (1995) 65(1): 101–17. 10.1016/0306-4522(94)00480-S 7753393

[210] Sasaki K Suzuki M Mieda M Tsujino N Roth B Sakurai T. Pharmacogenetic modulation of orexin neurons alters sleep/wakefulness states in mice. PLoS One (2011) 6:e20360. 10.1371/journal.pone.0020360 21647372 PMC3103553

